# Uncertainty quantification in high-dimensional linear models incorporating graphical structures with applications to gene set analysis

**DOI:** 10.1093/bioinformatics/btae541

**Published:** 2024-09-10

**Authors:** Xiangyong Tan, Xiao Zhang, Yuehua Cui, Xu Liu

**Affiliations:** School of Statistics and Data Science, Jiangxi University of Finance and Economics, Nanchang 330013, China; School of Data Science, The Chinese University of Hong Kong, Shenzhen 518172, China; Department of Statistics and Probability, Michigan State University, East Lansing, MI 48824, United States; School of Statistics and Management, Shanghai University of Finance and Economics, Shanghai 200433, China; Yunnan Key Laboratory of Statistical Modeling and Data Analysis, Yunnan University, Kunming 650500, China

## Abstract

**Motivation:**

The functions of genes in networks are typically correlated due to their functional connectivity. Variable selection methods have been developed to select important genes associated with a trait while incorporating network graphical information. However, no method has been proposed to quantify the uncertainty of individual genes under such settings.

**Results:**

In this paper, we construct confidence intervals (CIs) and provide *P*-values for parameters of a high-dimensional linear model incorporating graphical structures where the number of variables *p* diverges with the number of observations. For combining the graphical information, we propose a graph-constrained desparsified LASSO (least absolute shrinkage and selection operator) (GCDL) estimator, which reduces dramatically the influence of high correlation of predictors and enjoys the advantage of faster computation and higher accuracy compared with the desparsified LASSO. Theoretical results show that the GCDL estimator achieves asymptotic normality. The asymptotic property of the uniform convergence is established, with which an explicit expression of the uniform CI can be derived. Extensive numerical results indicate that the GCDL estimator and its (uniform) CI perform well even when predictors are highly correlated.

**Availability and implementation:**

An R package implementing the proposed method is available at https://github.com/XiaoZhangryy/gcdl.

## 1 Introduction

In recent years, the significance of high dimensional statistical inference has notably increased across various fields of application. Instances encompass functional magnetic resonance imaging, scrutiny of high-throughput genomic sequences, and image analysis. Under the sparsity constraint, many powerful methods have emerged during the past two decades, such as the least absolute shrinkage and selection operator (LASSO; Tibshirani 1996), the smoothly clipped absolute deviation (Fan and Li 2001), and the minimax concave penalty (Zhang 2010). In particular, the LASSO method has the advantage of being a convex problem, which has allowed the development of many efficient computational algorithms, such as least-angle regression (Efron *et al.* 2004), coordinate descent (Breheny and Huang 2011), and the alternating direction method of multipliers (Li *et al.* 2015). Zhao and Yu (2006), Van De Geer and Bühlmann (2009), and Bickel *et al.* (2009) pointed out that strong conditions must be imposed to guarantee good performance in estimation, variable selection, and prediction. It was shown that the LASSO does not perform well when covariates are highly correlated.

To overcome the problems mentioned above, many estimation procedures have been developed in recent years by making use of certain structures of the explanatory variables. For example, utilizing adaptive weights for penalizing each regression coefficient in the LASSO penalty, Zou (2006) proposed the adaptive LASSO and demonstrated its oracle properties. Yuan and Lin (2006) introduced the group LASSO to handle group structure. Zou and Hastie (2005) proposed the elastic net to deal with groups of correlated variables. Tibshirani *et al.* (2005) proposed the fused LASSO, which combines an $L_{1}$ penalty with an $L_{1}$ fusion penalty to account for some smoothness of the coefficients. Hebiri and Van De Geer (2011) introduced the smooth LASSO using an $L_{1}+L_{2}$ penalty.

Another approach incorporating network structure has been proposed to address the weakness of the LASSO. Auxiliary network information may be summarized using an undirected weighted graph G=(V,E,W), where V={1,…,p} is the set of vertices that correspond to the *p* predictors, E={u∼v} is the set of edges indicating that the predictors *u* and *v* are linked in the graph, and W is the set of weights of the edges. Li and Li (2008) proposed a genetic network-constrained regularization procedure for estimation and variable selection in linear regression models. Li and Li (2010) extended this work to the general problem of regression analysis when predictors are measured on an undirected graph. Combining the minimax concave penalty with a Laplacian matrix, Huang *et al.* (2011) proposed an estimator and showed that it has oracle properties. These existing literature mainly consider the estimation and selection of regression coefficients without quantifying the uncertainty of the coefficient estimation. Since practical scientists are more interested in getting *P*-values or confidence intervals (CIs) for regression coefficients, the purpose of this study is to perform statistical inference in high-dimensional linear models incorporating graphical structure.

A considerable amount of work has been done on testing for individual regression coefficients in a high-dimensional setup, representing a shift from variable selection to uncertainty quantification in high-dimensional data analyses. Meinshausen *et al.* (2009) proposed a sample-splitting method to obtain valid *P*-values in high-dimensional problems. This method can be easily extended to many other models. Bühlmann (2013) introduced a ridge projection method that can be regarded as a direct extension of low-dimensional ridge regression to the high-dimensional case. Van de Geer *et al.* (2014) introduced the desparsified LASSO (DL; see also Javanmard and Montanari 2014, Zhang and Zhang 2014). Caner and Kock (2018) extended this method to linear models with non-sub-Gaussian error and covariates by the conservative LASSO. Wang *et al.* (2018) proposed a novel desparsified procedure in high-dimensional linear models with heteroscedastic variance. The desparsified method has been extended to other specific models, e.g. quantile regression (Zhao *et al.* 2014), Gaussian graphical models (Ren *et al.* 2015), and inverse covariance estimation (Jankova and Van De Geer 2015).

The desparsified method may not work well for highly correlated predictors. A well-motivated example is gene set analysis in genomic studies (Wang *et al.* 2011, Maleki *et al.* 2020). Genes are functional units in most living organisms. Genes do not function alone; rather, they operate on a network basis to fulfill their joint tasks. Thus, it is critically important to assess the association of a network of correlated genes with a disease trait. Accumulative biological evidence have helped the establishment of various gene networks, such as the Kyoto Encyclopedia of Genes and Genomes (KEGG) pathways (Kanehisa and Goto 2000) and Gene Ontology (GO) terms (Ashburner *et al.* 2000). Genes belonging to the same KEGG pathway or GO term often function in a coordinated manner to implement common functions, and are often correlated due to complicated regulatory mechanisms. When assessing the association of a pathway with a phenotype (see the example in the real data analysis section 4), methods that ignore the graph information in a pathway may not work well and may lead to biased inference results. Incorporating prior graph information (e.g. obtained from the KEGG pathway database) is essential when assessing individual gene effects in a pathway.

In this work, we propose a new procedure that makes use of auxiliary network information in a high-dimensional linear model. We call the proposed approach the graph-constrained desparsified LASSO (GCDL) method. Specifically, we combine the LASSO and the Laplacian quadratic as the penalty function. GCDL uses the Laplacian quadratic penalty to encourage smoothness among coefficients associated with the correlated predictors. Simulation results show that our method performs better than existing methods and has competitive computational efficiency. The proposed work shows novelty in several aspects: (i) Our method can quantify the uncertainty of individual coefficients while incorporating graphical structures and is different from the current variable selection methods; (ii) it is computationally faster and has higher accuracy compared with the DL algorithm; (iii) we established the asymptotic property of uniform convergence, with which an explicit expression of uniform CI can be derived; and (iv) it is practically motivated, in particular in genomic analysis where the interest is to test the significance of individual genes that belong to a network. Such applications are common in genomic analyses.

The remainder of the paper is organized as follows. In Section 2, we propose a new DL estimation method that takes account of graph structure, and we investigate its asymptotic properties. In Sections 3 and 4, we report simulation studies and a real data analysis, respectively. In Section 5, we discuss our results. Finally, we give proofs of theorems and associated technical details in the Supplementary Materials file.

## 2 Materials and methods

### 2.1 Notation

Here, we summarize some standard notation employed throughout the paper. For an n×p random design matrix X and j∈{1,…,p}, define $X_{j}$ as the *j*th column of X. For i∈{1,…,n}, let $X_{i}$ be the *i*th row of X. For sets $D_{1}\in \{1,\ldots ,n\}$ and $D_{2}\in \{1,\ldots ,p\}$, let $X_{D_{1},D_{2}}$ denote the submatrix of X consisting only of the rows and columns indexed by $D_{1}$ and $D_{2}$, respectively. If $D_{1}=\{1,\ldots ,n\}$, we use $X_{D_{2}}$ as shorthand for the matrix $X_{D_{1},D_{2}}$. If $b={\left(b_{1},\ldots ,b_{p}\right)}^{T}$, we let $b_{D_{2}}$ be the modification of b that places zeros in all entries of b whose index does not belong to $D_{2}$. For any m×n matrix *A*, define $\left\|A{\|}_{\infty }=\max_{1\leq i\leq m,1\leq j\leq n}|A_{i,j}\right|$. For any symmetric matrix *B*, let $\Lambda_{\min}(B)$ and $\Lambda_{\max}(B)$ denote the smallest and largest eigenvalues of *B*, respectively. We denote by *c* a generic positive constant, which may take different values at different places.

### 2.2 The model

Suppose that a set of *n* independent samples ${\left(Y_{i},X_{i}\right)}_{i=1}^{n}$ has been collected from the following linear model:

(1) $$Y_{i}=X_{i}^{⊤}\beta +\varepsilon_{i},$$

where $X_{i}∼N(0,\Sigma )$ is a *p*-dimensional random vector, $\beta ={\left(\beta_{1},\ldots ,\beta_{p}\right)}^{⊤}\in R^{p}$ is an unknown but sparse vector, and $\varepsilon_{i}∼N(0,\sigma^{2})$. Without loss of generality, we assume $\Sigma_{j,j}=1$ for all j∈{1,…,p}. Let $Y={\left(Y_{1},\ldots ,Y_{n}\right)}^{⊤}$, $X={\left(X_{1},\ldots ,X_{n}\right)}^{⊤}$, and $\varepsilon =(\varepsilon_{1},\ldots ,\varepsilon_{n})$.

In this paper, we are interested in the statistical inference of $\beta_{j}$ in high-dimensional settings for j∈{1,…,p}, where *p* depends on *n* and diverges. Denote by $S_{0}:=\{j:\beta_{j}\neq 0\}$ the active set of variables, and by $s_{0}:=|S_{0}|$ its cardinality.

### 2.3 DL based on graph

Let *L* be a positive-semidefinite matrix that encodes the auxiliary information in an undirected weighted graph G=(V,E,W), where V={1,…,p} is the set of vertices that correspond to the *p* predictors, E={u∼v} is the set of edges indicating that the predictors *u* and *v* are linked in a graph, and W is a set of weights of edges. This graph Laplacian matrix *L* can be defined as

$$\begin{array}{c} L_{\left(u,v\right)}≜\{\begin{array}{cc} d_{u} & \text{if}\;u=v,\\ -w(u,v) & \text{if}\;u\;\text{and}\;v\;\text{are connected},\\ 0 & \text{otherwise}, \end{array} \end{array}$$

with w(u,v)≥0, where w(u,v) is the weight of edge e=(u∼v), and $d_{u}=\sum_{v∼u}w(u,v)$ is the degree of node *u*.

Let $\hat{\Sigma }=X^{⊤}X/n$ and $\lambda =(\lambda_{1},\lambda_{2})\geq 0$. We propose the penalized least squares (LS) estimator

(2) $$\hat{\beta }:=\hat{\beta }(\lambda_{1},\lambda_{2})=\underset{\beta \in R^{p}}{\text{argmin}}\left\{\frac{1}{2n}\|Y-X\beta {\|}_{2}^{2}+\lambda_{1}\|\beta {\|}_{1}+\frac{1}{2}\lambda_{2}\beta^{⊤}L\beta \right\}.$$

By the first-order condition of Equation (2), we have $\lambda_{1}\hat{z}+\lambda_{2}L\hat{\beta }=X^{⊤}(Y-X\hat{\beta })/n$, and consequently

(3) $$\hat{\Sigma }(\hat{\beta }-\beta )+\lambda_{1}\hat{z}+\lambda_{2}L\hat{\beta }=X^{⊤}\varepsilon /n,$$

where $\hat{z}$ is a *p*-dimensional vector with $\|\hat{z}{\|}_{\infty }\leq 1$ and with *j*th element ${\hat{z}}_{j}=\text{sign}({\hat{\beta }}_{j})$ if ${\hat{\beta }}_{j}\neq 0$, where sign(x) is the sign function taking values 1, 0, or −1 for x>0, x=0, or x<0, respectively.

To isolate $\hat{\beta }-\beta$ from Equation (3), we need to invert $\hat{\Sigma }$. However, when p>n, $\hat{\Sigma }$ is not invertible. Suppose that $\hat{\Theta }$ is a reasonable approximation of the inverse of $\hat{\Sigma }$. Then, multiplying $\hat{\Theta }$ into both sides of Equation (3), we have

(4) $$\begin{array}{c} \hat{\beta }-\beta +\lambda_{1}{\hat{\Theta }}^{⊤}\hat{z}+\lambda_{2}{\hat{\Theta }}^{⊤}L\hat{\beta }={\hat{\Theta }}^{⊤}X^{⊤}\varepsilon /n-\Delta_{n}/\sqrt{n}, \end{array}$$

where $\Delta_{n}=\sqrt{n}({\hat{\Theta }}^{⊤}\hat{\Sigma }-I)(\hat{\beta }-\beta )$. We will show in Theorem 2 that $\Delta_{n}$ is asymptotically negligible under certain assumptions.

Define $\Theta =\Sigma^{-1}$. By Equation (4), we define the GCDL as

$$\hat{b}={\left({\hat{b}}_{1},\ldots ,{\hat{b}}_{p}\right)}^{⊤}=\hat{\beta }+\frac{1}{n}{\hat{\Theta }}^{⊤}X^{⊤}(Y-X\hat{\beta }),$$

which implies

(5) $$\begin{array}{c} {\hat{b}}_{j}-\beta_{j}={\hat{\Theta }}_{j}^{⊤}X^{⊤}\varepsilon /n-{\left(\hat{\Sigma }{\hat{\Theta }}_{j}-e_{j}\right)}^{⊤}(\hat{\beta }-\beta )\\ =\Theta_{j}^{⊤}X^{⊤}\varepsilon /n+{\left({\hat{\Theta }}_{j}-\Theta_{j}\right)}^{⊤}X^{⊤}\varepsilon /n-{\left(\hat{\Sigma }{\hat{\Theta }}_{j}-e_{j}\right)}^{⊤}(\hat{\beta }-\beta ), \end{array}$$

where $\Theta_{j}$, ${\hat{\Theta }}_{j}$, and $e_{j}$ are the *j*th columns of Θ, $\hat{\Theta }$, and $I_{p}$, respectively.

In the next section, we use node-wise regression to estimate $\hat{\Theta }$, which plays a crucial role in the inference step.

### 2.4 Constructing the approximate inverse of the Gram matrix: $\hat{\Theta }$

In this subsection, we propose two methods to construct the approximate inverse of the Gram matrix $\hat{\Theta }$. Let $D_{j}=\{k:L_{j,k}\neq 0,k\neq j\}$, $d_{j}=|D_{j}|$, $R_{j}=\{1,\ldots ,p\}-D_{j}-\{j\}$, $r_{j}=|R_{j}|$, and $d=\max\{d_{1},\ldots ,d_{p}\}$ for j∈{1,…,p} and k∈{1,…,p}.

#### 2.4.1 Node-wise regression: *d* is small

For the first method to construct $\hat{\Theta }$, we borrow an idea from Meinshausen and Bühlmann (2006) and use node-wise regression to estimate $\hat{\Theta }$. In our theoretical analysis, we focus only on this method. For each j=1,…,p, let

(6) $${\hat{\gamma }}_{j}:=\underset{\gamma \in R^{d_{j}}}{\text{argmin}}\frac{1}{n}\|X_{j}-X_{D_{j}}\gamma {\|}_{2}^{2},$$

and

$${\hat{\tau }}_{j}^{2}:=\frac{1}{n}\|X_{j}-X_{D_{j}}{\hat{\gamma }}_{j}{\|}_{2}^{2}.$$

If $D_{j}=\emptyset$, then we set ${\hat{\gamma }}_{j}=0$ and ${\hat{\tau }}_{j}^{2}=\|X_{j}{\|}_{2}^{2}/n.$ Define ${\hat{\Theta }}_{j}={\left({\hat{\Theta }}_{1,j},\ldots ,{\hat{\Theta }}_{p,j}\right)}^{T}$ as

$$\begin{array}{c} {\hat{\Theta }}_{j,j}=1/{\hat{\tau }}_{j}^{2},\;\;j\in \{1,\ldots ,p\},\\ {\hat{\Theta }}_{k,j}=\{\begin{array}{cc} -{\hat{\gamma }}_{k,j}/{\hat{\tau }}_{j}^{2}, & k\neq j,\;k\in D_{j},\\ 0, & k\neq j,\;k\notin D_{j}, \end{array} \end{array}$$

where ${\hat{\gamma }}_{k,j}$ is the *k*-th element of ${\hat{\gamma }}_{j}$. Then, $\hat{\Theta }=({\hat{\Theta }}_{1},\ldots ,{\hat{\Theta }}_{p})$.

Since there is no requirement of selection of the tuning parameter in equality (6), the proposed method enjoys the advantage of faster computation and higher accuracy compared with the DL.

For each j=1,…,p, define $\gamma_{j}$ as

$$\gamma_{j}:=\underset{\gamma \in R^{d_{j}}}{\text{argmin}}E{\left[X_{j}-X_{D_{j}}\gamma \right]}^{2},$$

and the error $\eta_{j}:=X_{j}-X_{D_{j}}\gamma_{j}$. It can be observed that $\tau_{j}^{2}:=E(\eta_{j}^{T}\eta_{j})/n=1/\Theta_{j,j}$.

#### 2.4.2 Node-wise regression: *d* is large

In practice, however, *d* may be too large. For example, for our real data analysis in Section 4, the sample size is p=70, and d=58. This violates Condition (C5) in Section 2.5. Thus, we extend the first method and use the following procedure to estimate $\hat{\Theta }$.

For each j=1,…,p, and for some constant integer *k*, let

(7) $$\begin{array}{c} {\hat{\gamma }}_{j}:=\{\begin{array}{cc} \underset{\gamma \in R^{d_{j}}}{\text{argmin}}\frac{1}{n}\|X_{j}-X_{D_{j}}\gamma {\|}_{2}^{2} & \text{if}\;d_{j}\leq k,\\ \underset{\gamma \in R^{d_{j}}}{\text{argmin}}\frac{1}{2n}\|X_{j}-X_{D_{j}}\gamma {\|}_{2}^{2}+\lambda_{j}\|\gamma {\|}_{1} & \text{if}\;d_{j}>k, \end{array} \end{array}$$

and

$$\begin{array}{c} {\hat{\tau }}_{j}^{2}:=\{\begin{array}{cc} \frac{1}{n}\|X_{j}-X_{D_{j}}{\hat{\gamma }}_{j}{\|}_{2}^{2} & \text{if}\;d_{j}\leq k,\\ \frac{1}{n}\|X_{j}-X_{D_{j}}{\hat{\gamma }}_{j}{\|}_{2}^{2}+\lambda_{j}\|{\hat{\gamma }}_{j}{\|}_{1} & \text{if}\;d_{j}>k. \end{array} \end{array}$$

We define ${\hat{\Theta }}_{j}={\left({\hat{\Theta }}_{1,j},\ldots ,{\hat{\Theta }}_{p,j}\right)}^{T}$ as

$$\begin{array}{c} {\hat{\Theta }}_{j,j}=1/{\hat{\tau }}_{j}^{2},\;\;j\in \{1,\ldots ,p\},\\ {\hat{\Theta }}_{k,j}=\{\begin{array}{cc} -{\hat{\gamma }}_{k,j}/{\hat{\tau }}_{j}^{2}, & k\neq j,\;k\in D_{j},\\ 0, & k\neq j,\;k\notin D_{j}. \end{array} \end{array}$$

Then, $\hat{\Theta }=({\hat{\Theta }}_{1},\ldots ,{\hat{\Theta }}_{p})$.

In Section 3.2, we will demonstrate the validity of this procedure by simulations.

### 2.5 Main theoretical results

#### 2.5.1 CIs for preconceived parameters

The following conditions are required for the asymptotic guarantees:

(C1) Let $L=Q^{⊤}Q$ and $K_{n}=\hat{\Sigma }+\lambda_{2}Q^{⊤}Q$. There is a constant $\phi_{n}>0$ such that, for any $\tilde{\beta }\in R^{p}$ that satisfies $\|{\tilde{\beta }}_{S_{0}^{c}}{\|}_{1}\leq 4\|{\tilde{\beta }}_{S_{0}}{\|}_{1}$, we have ${\tilde{\beta }}^{⊤}K_{n}\tilde{\beta }\geq \phi_{n}\|{\tilde{\beta }}_{S_{0}}{\|}_{2}^{2}$.
(C2) $\lambda_{1}≍\sqrt{\;\text{log}\;p/n}$ and $\lambda_{2}=0.1\lambda_{1}/\|L\beta {\|}_{\infty }$. Where ≍ means asymptotically equal.
(C3) The complement of the set $E=\{2\|X^{⊤}\varepsilon {\|}_{\infty }/n\leq \lambda_{1}\}$ satisfies $P(E^{C})=O(p^{-c})$ for some positive constant *c*.
(C4) $\Lambda_{\min}(\Sigma )$ is bounded away from zero. $\Lambda_{\max}(\Sigma )$ is bounded from above.
(C5) $d^{2}\;\text{log}\;d=o(n/\;\text{log}\;p)$ and $s_{0}=o(\sqrt{n}/\;\text{log}\;p)$.
(C6) For any j∈{1,…,p}, $\Lambda_{\min}((1/n)X_{D_{j}}^{⊤}X_{D_{j}})$ is bounded away from zero.

Remark 1. *Conditions (C1) and (C2) involve a set of linear inequalities, which were imposed in Hebiri and Van De Geer (2011*)*. It is not hard to see that*$\phi_{n}>\phi_{0}$*, where*$\phi_{0}$*corresponds to the LASSO, i.e.*$\lambda_{2}=0$*. Condition (C3) is imposed on the error tail distribution and can easily be derived using the classical Gaussian tail probability bound. Condition (C4) implies that*$1/\Theta_{j,j}\geq \Lambda_{\min}(\Sigma )$*stays away from zero. Condition (C5) restricts the growth rates of d and*$s_{0}$*. The order of*$s_{0}$*is a common condition that has been used by Van de Geer et al. (2014) and Zhang and Zhang (2014). The order of d is a mild condition that makes*${\left({\hat{\Theta }}_{j}-\Theta_{j}\right)}^{⊤}X^{⊤}\varepsilon /n=o_{p}(n^{-1/2})$*and*${\left(\hat{\Sigma }{\hat{\Theta }}_{j}-e_{j}\right)}^{⊤}(\hat{\beta }-\beta )=o_{p}(n^{-1/2})$*. For example, when*$d=O(\sqrt{n}/\;\text{log}\;p)$*, Condition (C5) is satisfied, which is used in Van de Geer et al. (2014). Condition (C6) is used to ensure that node-wise regression is available. These conditions are quite mild and can be verified in practice. To show the utility of the proposed method, we provided verifications of the conditions in the real data analysis (see the details in Web Appendix C of the Supplementary Material).*

We now present the main theorems. The proofs are given in Web Appendix A of the Supplementary Material.

Theorem 1. *Let*${\tilde{\lambda }}_{j}=\sqrt{2(n-d_{j})\;\text{log}(p-d_{j}-1)/(c\Theta_{j,j}n^{2})}$*for some constant c. Then, with probability at least*$1-2{\left(p-d_{j}-1\right)}^{-1}$*, we have*

$$\|\hat{\Sigma }{\hat{\Theta }}_{j}-e_{j}{\|}_{\infty }\leq {\tilde{\lambda }}_{j}/{\hat{\tau }}_{j}^{2}.$$

Theorem 1 provides the non-asymptotic upper bound for $\|\hat{\Sigma }{\hat{\Theta }}_{j}-e_{j}{\|}_{\infty }$, which is an important result to prove the asymptotic normality of ${\hat{b}}_{j}$, as stated in Theorem 2.

Theorem 2. *Assuming that Conditions (C1)–(C6) hold, we have*

$$\sqrt{n}({\hat{b}}_{j}-\beta_{j})\overset{L}{\to }(0,\sigma^{2}\Theta_{j}^{⊤}\Sigma \Theta_{j})\;\;\mathrm{and}\;\;|{\hat{\Theta }}_{j}^{⊤}\hat{\Sigma }{\hat{\Theta }}_{j}-\Theta_{j}^{⊤}\Sigma \Theta_{j}|=o_{p}(1),$$

*where*$\overset{L}{\to }$*denotes convergence in distribution.*

Remark 2. *ByTheorem 2, we can conduct a Wald-type test for*$\beta_{j}$*. Furthermore, we can easily extend this to joint inference on any fixed number of elements of*β*, although we shall not pursue this extension further here.*

Remark 3. *To construct a CI for*$\beta_{j}$*, an estimator*${\hat{\sigma }}^{2}$*of the error variance*$\sigma^{2}$*is needed. In this paper, we use the refitted cross-validation procedure (Fan et al. 2012) to estimate*$\sigma^{2}$*. The CI for*$\beta_{j}$*for*j∈{1,…,p}*can be obtained byTheorem 2. The detailed algorithm is summarized in Algorithm 1.*

Remark 4. *The findings fromTheorem 2 suggest the asymptotic distribution is independent of the graph Laplacian matrix L. Theorem 1 establishes that the rate of convergence for*$\|\hat{\Sigma }{\hat{\Theta }}_{j}-e_{j}{\|}_{\infty }$*is*$O_{p}\left(\sqrt{\frac{n-d_{j}}{n}\frac{\;\text{log}(p-d_{j}-1)}{\;\text{log}\;p}}\sqrt{\;\text{log}\;p/n}\right)$*, which signifies a more rapid convergence compared to the DL (Van de Geer et al. 2014), whose rate is*$O_{p}\left(\sqrt{\;\text{log}\;p/n}\right)$*. Furthermore*, *Lemma S.3 in the Supplementary Material demonstrates that*$\|{\hat{\Theta }}_{j}-\Theta_{j}{\|}_{1}=O_{p}\left(\sqrt{\frac{\;\text{log}\;d}{\;\text{log}\;p}}d\sqrt{\;\text{log}\;p/n}\right)$*, which also exhibits a more rapid convergence than the DL methods, whose rate is*$O_{p}\left(d\sqrt{\;\text{log}\;p/n}\right)$*. Consequently, compared to the DL without graph structures, both bias terms*$\left|\sqrt{n}{\left(\hat{\Sigma }{\hat{\Theta }}_{j}-e_{j}\right)}^{⊤}(\hat{\beta }-\beta )\right|$*and*$\left|\sqrt{n}{\left({\hat{\Theta }}_{j}-\Theta_{j}\right)}^{⊤}X^{⊤}\varepsilon /n\right|$*inEquation (5) exhibit faster convergence toward zero. These observations indicate that the GCDL method exhibits better performance under finite samples.*

Algorithm 1: Computing algorithm

**Require: Input:** ${\left\{X_{i},Y_{i}\right\}}_{i=1}^{n}$

 **Output:** The confidence interval (CI) of $\beta_{j}$ for j∈{1,…,p}

 **Procedure**

 1. Calculate the initial estimator of β by (2);

 2. Calculate the ${\hat{\gamma }}_{j}$ by (6) and then construct $\hat{\Theta }$;

 3. Calculate the desparsified LASSO estimator by $\hat{b}=\hat{\beta }+\frac{1}{n}{\hat{\Theta }}^{⊤}X^{⊤}(Y-X\hat{\beta }).$

 4. Calculate the CI of $\beta_{j}$ by $[{\hat{b}}_{j}-n^{-1/2}z_{1-\alpha /2}{\hat{\sigma }}_{j},{\hat{b}}_{j}+n^{-1/2}z_{1-\alpha /2}{\hat{\sigma }}_{j}],$ where ${\hat{\sigma }}_{j}=\sqrt{{\hat{\sigma }}^{2}{\hat{\Theta }}_{j}^{⊤}\hat{\Sigma }{\hat{\Theta }}_{j}}$.

 **End procedure**

#### 2.5.2 Simultaneous CI

Theorem 2 allows us to construct CI for individual coefficient $\beta_{j}$. To achieve the uniform convergence of $\beta_{j}$’s estimator instead of pointwise, we construct simultaneous CIs in the following Theorem.

Theorem 3. *Let*$B_{l_{0}}(s_{0})=\{\beta :\|\beta {\|}_{0}\leq s_{0}\}$*. Assuming Conditions (C1)–(C6) hold, for all*j∈{1,…,p}*, we have*

1. $\underset{t\in R}{\sup}\underset{\beta \in B_{l_{0}}(s_{0})}{\sup}|P(\sqrt{n}({\hat{b}}_{j}-\beta_{j})/{\hat{\sigma }}_{j}\leq t)-\Phi (t)|\to 0,$
2. $\underset{n\to \infty }{\lim}\underset{\beta \in B_{l_{0}}(s_{0})}{\inf}P(\beta_{j}\in [{\hat{b}}_{j}-n^{-1/2}z_{1-\alpha /2}{\hat{\sigma }}_{j},{\hat{b}}_{j}+n^{-1/2}z_{1-\alpha /2}{\hat{\sigma }}_{j}])=1-\alpha ,$
3. $\underset{\beta \in B_{l_{0}}(s_{0})}{\sup}\text{diam}[{\hat{b}}_{j}-n^{-1/2}z_{1-\alpha /2}{\hat{\sigma }}_{j},{\hat{b}}_{j}+n^{-1/2}z_{1-\alpha /2}{\hat{\sigma }}_{j}]=O_{p}(n^{-1/2}),$

*where*${\hat{\sigma }}_{j}=\sqrt{{\hat{\sigma }}^{2}{\hat{\Theta }}_{j}^{⊤}\hat{\Sigma }{\hat{\Theta }}_{j}}$, $z_{1-\alpha /2}$*is the*1−α/2*quantile of the standard normal distribution and*diam([a,b])=b−a*is the length of an interval [a, b].*

Remark 5. *Theorem 3 reveals that the CI*$\left[{\hat{b}}_{j}-n^{-1/2}z_{1-\alpha /2}{\hat{\sigma }}_{j},{\hat{b}}_{j}+n^{-1/2}z_{1-\alpha /2}{\hat{\sigma }}_{j}\right]$*is asymptotically uniform.*

#### 2.5.3 Global test

One may be interested in test problem $H_{0}:\beta =0$ versus $H_{1}:\beta \neq 0$, which motivates us to consider the test statistic $T_{n}=\underset{1\leq j\leq p}{\max}T_{j}^{2}$, where $T_{j}=\sqrt{n}({\hat{b}}_{j}-\beta_{j})/\sqrt{{\hat{\sigma }}^{2}{\hat{\Theta }}_{j}^{⊤}\hat{\Sigma }{\hat{\Theta }}_{j}}$, j=1,…,p.

Theorem 4. *Assume Conditions (C1)–(C4) and (C6) inTheorem 2 hold, and*$\text{log}\;p=O(n^{\gamma })$*for some*0<γ<1/5,$s_{0}=o(\sqrt{n}/{\;\text{log}\;}^{3/2}p)$,$d=o(\sqrt{n}/{\;\text{log}\;}^{5/2}p)$*. Then for any given*x∈R*, we have*

$$P(T_{n}-2\;\text{log}\;p+\text{log}\;\;\text{log}\;p\leq x)\to \;\text{exp}(-\text{exp}(-x/2)/\sqrt{\pi }),\text{as}\;(n,p)\to \infty .$$

Remark 6. *Let*$C_{\alpha }=2\;\text{log}\;p-\text{log}\;\;\text{log}\;p+q_{\alpha }$*, where*$q_{\alpha }=-\text{log}(\pi )-2\;\text{log}\;\;\text{log}\;{\left(1-\alpha \right)}^{-1}$*is the*1−α*quantile of the distribution with the cumulative distribution function*$\text{exp}(-\text{exp}(-x/2)/\sqrt{\pi })$. *Theorem 4 implies that the null hypothesis*$H_{0}:\beta =0$*is rejected if*$\underset{1\leq j\leq p}{\max}n{\hat{b}}_{j}^{2}/{\hat{\sigma }}_{j}^{2}\geq C_{\alpha }$.

## 3 Simulation studies

In this section, we conduct Monte Carlo simulations to assess the finite-sample performance of the proposed method. We consider two scenarios according to whether or not *d* satisfies the condition $d^{2}\;\text{log}\;d=o(n/\;\text{log}\;p)$.

We consider the linear regression model

(8) $$Y_{i}=X_{i}^{⊤}\beta +\varepsilon_{i},$$

where the random error $\varepsilon_{i}$ is independently generated from N(0,1). The covariates $X_{i}={\left(X_{i1},\ldots ,X_{ip}\right)}^{⊤}$ are randomly generated from a *p*-dimensional normal distribution N(0,Σ). The findings presented in Web Appendix E demonstrate a notable non-sensitivity in the edge weights. Consequently, we construct the Laplacian matrix *L* according to a graph with all edge weights equal to 1. We set $S_{0}=\{1,\ldots ,15\}$ and all simulation results are based on 1000 replications.

### 3.1 Scenario I

Example 1: We generate a dataset from model (8) with n=200 and p=500. Set $\beta_{j}=3$ for $j\in S_{0}$ and 0 otherwise. We construct five groups of correlated variables: $\Sigma_{j,j}=1$ for each j∈{1,…,p}; for i≠j, $\Sigma_{i,j}=\frac{1}{1.01}$ if (i,j) belongs to {1,2,3}×{1,2,3}, {4,5,6}×{4,5,6}, {7,8,9}×{7,8,9}, {10,11,12}×{10,11,12}, or {13,14,15}×{13,14,15}, and $\Sigma_{i,j}=0$ otherwise. This example is similar to that used in Hebiri and Van De Geer (2011).

Example 2: The setup is similar to Example 1, with the exception that $\Sigma_{i,j;i\neq j}=0.5$ when (i,j) belongs to {1,2,3}×{1,2,3}, {4,5,6}×{4,5,6}, {7,8,9}×{7,8,9}, {10,11,12}×{10,11,12}, or {13,14,15}×{13,14,15}, and is zero otherwise. For β, we let $\beta_{j}=1$ for $j\in S_{0}$ and zero otherwise.

According to the definition of *d*, we have d=2 in Scenario I. We first compare the empirical coverage probabilities constructed by the proposed procedure with the DL estimator (Zhang and Zhang 2014), the desparsified ridge (DR) estimator (Bühlmann 2013), and the LS estimator. In the case of the LS estimator, the true zero coefficients are known and no shrinkage is applied. For convenience in the description, define ${\hat{b}}_{j}^{\mathrm{GCDL}}$ to mean “based on the GCDL method,” with ${\hat{b}}_{j}^{DL}$, ${\hat{b}}_{j}^{DR}$, and ${\hat{\beta }}_{j}^{LS}$ being defined analogously. According to Theorem 2, the 95% CI $CI_{j}^{\mathrm{GCDL}}$ of $\beta_{j}$ based on the GCDL estimator is constructed as follows:

$$CI_{j}^{\mathrm{GCDL}}={\hat{b}}_{j}^{\mathrm{GCDL}}\pm \frac{1.96}{\sqrt{n}}\hat{\sigma }\sqrt{{\hat{\Theta }}_{j}^{⊤}\hat{\Sigma }{\hat{\Theta }}_{j}}.$$

For the DL and DR estimators, the method for constructing the 95% CIs of $\beta_{j}$ ($CI_{j}^{DL}$ and $CI_{j}^{DR}$) is implemented in the *R* package *hdi* (Dezeure *et al.* 2015). For the LS estimator, the 95% CI of $\beta_{j},j\in S_{0}$ is constructed as follows:

$$CI_{j}^{LS}={\hat{\beta }}_{j}^{LS}\pm \frac{1.96}{\sqrt{n}}{\hat{\sigma }}_{LS}\sqrt{{\hat{\Theta }}_{j,j}^{LS}}\;,$$

where ${\hat{\sigma }}_{LS}^{2}={\left(n-|S_{0}|\right)}^{-1}\sum_{i=1}^{n}{\left(Y_{i}-X_{i,S_{0}}^{⊤}{\hat{\beta }}^{LS}\right)}^{2}$, ${\hat{\Theta }}^{LS}={\left(n^{-1}\sum_{i=1}^{n}X_{i,S_{0}}X_{i,S_{0}}^{⊤}\right)}^{-1}$, and ${\hat{\beta }}_{j}^{LS}$ is the *j*-th element of ${\hat{\beta }}^{LS}={\left(\sum_{i=1}^{n}X_{i,S_{0}}X_{i,S_{0}}^{⊤}\right)}^{-1}X_{i,S_{0}}^{⊤}Y$.

We compute the empirical coverage probability $CP_{j}^{\mathrm{GCDL}}=P_{n}(\beta_{j}\in CI_{j}^{\mathrm{GCDL}})$, where $P_{n}$ denotes the empirical probability based on 1000 realizations. The coverage probabilities $CP_{j}^{DL}$, $CP_{j}^{DR}$, and $CP_{j}^{LS}$ are defined analogously. Also, we provide the average lengths $AL_{j}^{\mathrm{GCDL}}$, $AL_{j}^{DL}$$AL_{j}^{DR}$, and $AL_{j}^{LS}$ of the CIs $CI_{j}^{\mathrm{GCDL}}$, $CI_{j}^{DL}$, $CI_{j}^{DR}$, and $CI_{j}^{LS}$, respectively, for j=1,…,p. Define $CP_{S_{0}}^{\mathrm{GCDL}}=\{CP_{j}^{\mathrm{GCDL}}:j\in S_{0}\}$. $CP_{S_{0}^{c}}^{\mathrm{GCDL}}$, $CP_{S_{0}}^{DL}$, $CP_{S_{0}^{c}}^{DL}$, $CP_{S_{0}}^{DR}$, $CP_{S_{0}^{c}}^{DR}$, and $CP_{S_{0}}^{LS}$ are defined analogously.

**Figure 1.**
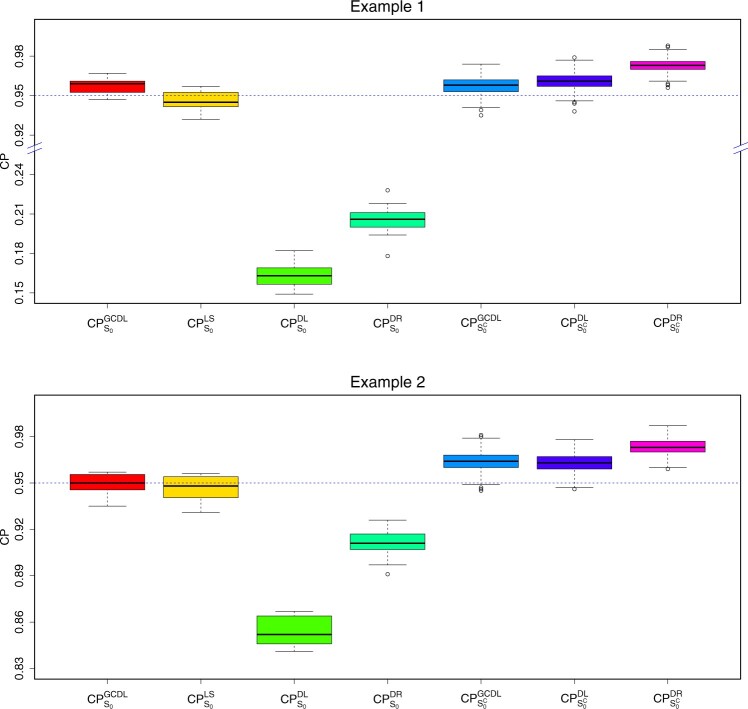
The 95% coverage probabilities computed using four methods: GCDL, LS, DL, and DR. The *y*-axis in the upper panel is truncated.

Under two settings, the coverage probabilities in Fig. 1 demonstrate that the $CP_{j}^{\mathrm{GCDL}},j=1,\ldots ,500,$ are approximately 95%, which is comparable to the result from the LS estimator, whereas $CP_{j}^{DL},j\in S_{0}$ falls below 20% in Example 1 and 90% in Example 2. We obtain a similar result for the DR estimator. Thus, our method performs much better than the DL and DR methods, especially in the case of highly correlated predictors.

We summarize the results for the coverage probabilities and interval lengths by taking averages across coordinates on $S_{0}$ and $S_{0}^{c}$; i.e. we define

$$\mathrm{AveC}P_{S_{0}}=\frac{1}{s_{0}}\sum_{j\in S_{0}}CP_{j},\;\mathrm{AveC}P_{S_{0}^{c}}=\frac{1}{p-s_{0}}\sum_{j\in S_{0}^{c}}CP_{j}$$

and

$$\mathrm{AveA}L_{S_{0}}=\frac{1}{s_{0}}\sum_{j\in S_{0}}AL_{j},\;\mathrm{AveA}L_{S_{0}^{c}}=\frac{1}{p-s_{0}}\sum_{j\in S_{0}^{c}}AL_{j}.$$

Table 1 summarizes the average coverage probabilities and interval lengths. For the zero regression coefficients in Examples 1 and 2, although the average coverage probabilities are almost the same, GCDL tends to produce much narrower CIs. It is interesting to note that DL tends to produce smaller average CIs for the nonzero regression coefficients in Examples 1 and 2, but at the cost of lower coverage probabilities. For zero regression coefficients, the DR method produces larger coverage probabilities and larger average CIs in Example 2. Furthermore, the 90% and 99% coverage probabilities and interval lengths are provided in Web Appendix D.

**Table 1.** The 95% average coverage probabilities and interval lengths.

		$\mathrm{AveC}P_{S_{0}}$	$\mathrm{AveC}P_{S_{0}^{c}}$	$\mathrm{AveA}L_{S_{0}}$	$\mathrm{AveA}L_{S_{0}^{c}}$
Example 1	GCDL	0.9569	0.9575	2.3003	0.2785
	LS	0.9464		2.3737	
	DL	0.1634	0.9609	0.3310	0.3122
	DR	0.2057	0.9731	0.4080	0.4023
Example 2	GCDL	0.9494	0.9639	0.3430	0.2785
	LS	0.9468		0.3540	
	DL	0.8546	0.9629	0.3305	0.3157
	DR	0.9104	0.9733	0.4193	0.4036

**Figure 2.**
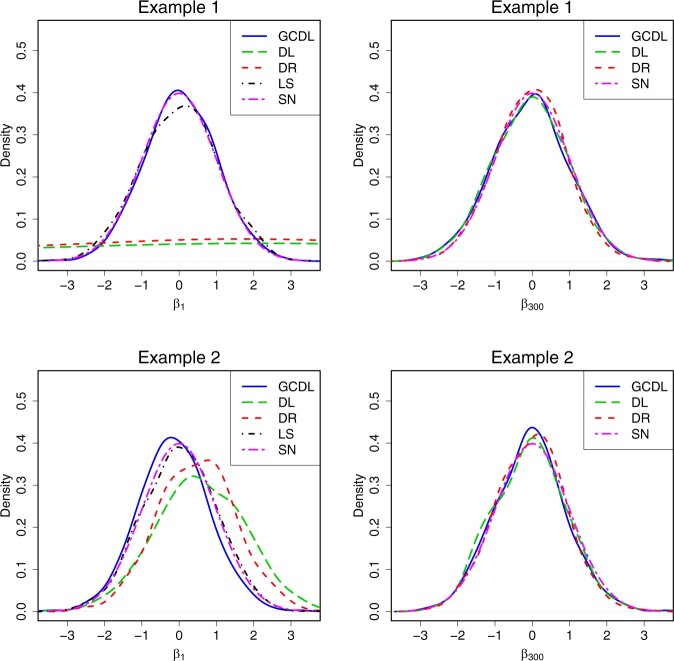
Empirical densities of ${\hat{b}}_{j}^{\mathrm{GCDL}}$, ${\hat{b}}_{j}^{DL}$, ${\hat{b}}_{j}^{DR}$, and ${\hat{\beta }}_{1}^{LS}$ for j=1 and 300 based on 1000 realizations. “SN” means standard normal.

We empirically verify the asymptotic normality of ${\hat{b}}_{j}^{\mathrm{GCDL}}$ in Theorem 2. We compute ${\hat{b}}_{j}^{\mathrm{GCDL}}$, j=1,…,p, based on 1000 realizations, and then plot their empirical densities. Figure 2 only shows the results for j=1 and 300.

In both settings, Fig. 2 confirms the asymptotic normality of the proposed method. Specifically, after centering and standardization, the distribution of ${\hat{b}}_{j}^{\mathrm{GCDL}}$ is close to the standard normal distribution for j=1 and 300, but the distribution of ${\hat{b}}_{j}^{DL}$ and ${\hat{b}}_{j}^{DR}$ is far from the standard normal distribution when j=1. Furthermore, when the regression coefficient is zero, the empirical densities of the four estimators are very close.

**Figure 3.**
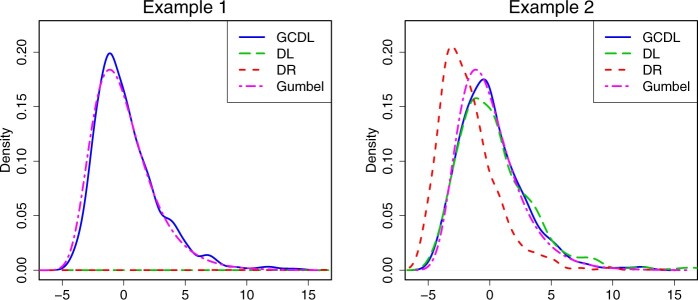
Empirical densities of $T_{n}^{\mathrm{GCDL}}$, $T_{n}^{DL}$, and $T_{n}^{DR}$ based on 1000 realizations.

We verify numerically the asymptotic distribution of $T_{n}$ in Theorem 4. Denote by $T_{n}^{\mathrm{GCDL}}$, $T_{n}^{DL}$ and $T_{n}^{DR}$ the test statistics based on the method GCDL, DL and DR, respectively. Figure 3 confirms the asymptotic distribution of the proposed method. Specifically, the distribution of $T_{n}^{\mathrm{GCDL}}$ is close to the Gumbel distribution with the cumulative distribution function $\text{exp}\;\left(-\frac{1}{\sqrt{\pi }}\text{exp}(-x/2)\right).$ But the distribution of $T_{n}^{DL}$ and $T_{n}^{DR}$ is far from the Gumbel distribution, especially in the case of highly correlated predictors.

### 3.2 Scenario II

We propose a new procedure, called adjusted GCDL (AGCDL). The main difference between this method and GCDL is that AGCDL uses Equation (7) to estimate $\hat{\Theta }$, whereas GCDL uses Equation (6). In this subsection, we will demonstrate the validity of AGCDL.

Example 3: We generate a dataset from model (8) with n=200 and p=200. For β, we set $\beta_{j}=3$ for $j\in S_{0}$ and zero otherwise. Moreover, $\Sigma_{j,j}=1$ for every j∈{1,…,p}; for i≠j, $\Sigma_{i,j}=\frac{1}{1.01}$ when (i,j) belongs to {1,2,3}×{1,2,3}, {4,5,6}×{4,5,6}, {7,8,9}×{7,8,9}, {10,11,12}×{10,11,12}, or {13,14,15}×{13,14,15}; for j≠k, $\Sigma_{j,k}=\text{exp}(-|j-k|)$ when (j,k) belongs to {16,…,200}×{16,…,200} and is zero otherwise.

Note that we need to determine the value of the integer *k* in Equation (7); the design indicates that $d_{j}\leq 2$ for j=1,…,15 and $d_{j}\geq 183$ for j=16,…,200. Thus, we choose k=2 in this simulation study.

**Figure 4.**
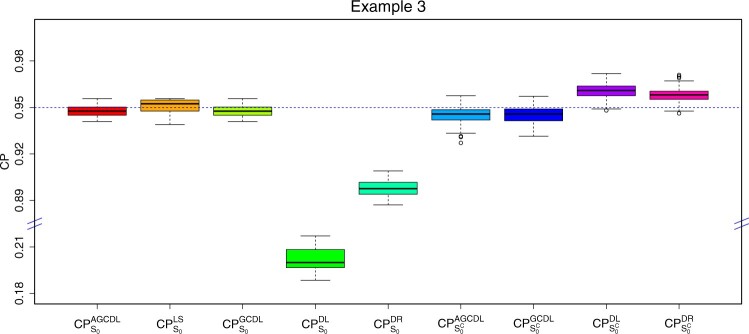
Coverage probabilities computed using five methods: AGCDL, GCDL, DL, DR, and LS. The *y*-axis is truncated.

**Figure 5.**
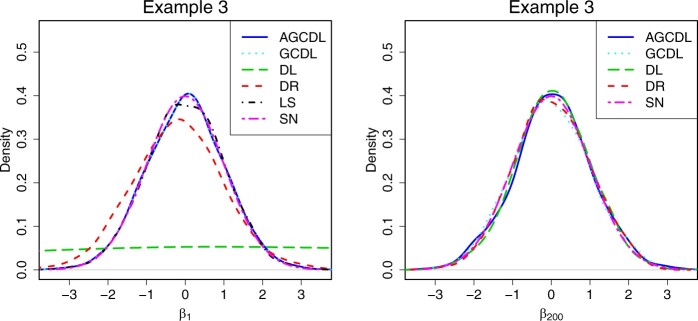
Empirical densities of ${\hat{b}}_{j}^{\mathrm{AGCDL}}$, ${\hat{b}}_{j}^{\mathrm{GCDL}}$, ${\hat{b}}_{j}^{DL}$, ${\hat{b}}_{j}^{DR}$, and ${\hat{\beta }}_{1}^{LS}$ for j=1 and 200 based on 1000 realizations. “SN” means standard normal.

Figure 4 shows that, for the empirical coverage probabilities of the nonzero coefficients, the DL and DR estimators provide such poor coverage (less than 22% and 91%, respectively) that they may not be good enough, while AGCDL and GCDL still have approximately 95% coverage. For the nonzero regression coefficients, Table 2 shows that DL has the narrowest average CI, but also the lowest average coverage probabilities. For the zero regression coefficients, GCDL has the longest CI, which indicates that GCDL does not perform well when *d* is large, whereas AGCDL achieves the narrowest CI. In Web Appendix D, we provide the 90% and 99% coverage probabilities along with their interval lengths. Figure 5 shows that the standardized distributions of ${\hat{b}}_{j}^{\mathrm{GCDL}}$ and ${\hat{b}}_{j}^{\mathrm{AGCDL}}$ are close to the standard normal distribution for j=1 and 200. However, the DL and DR estimators fail to converge when j=1. The above results demonstrate the validity of AGCDL.

**Table 2.** The 95% coverage probabilities and average interval lengths.

		$\mathrm{AveC}P_{S_{0}}$	$\mathrm{AveC}P_{S_{0}^{c}}$	$\mathrm{AveA}L_{S_{0}}$	$\mathrm{AveA}L_{S_{0}^{c}}$
Example 3	AGCDL	0.9477	0.9451	2.2903	0.2831
	LS	0.9509		2.3648	
	GCDL	0.9477	0.9455	2.2903	1.1570
	DL	0.2015	0.9608	0.3866	0.3251
	DR	0.8980	0.9580	1.0918	0.8133

Similarly, denote by $T_{n}^{\mathrm{AGCDL}}$ the test statistic based on the AGCDL method. Figure 9 in Web Appendix D confirms the asymptotic distribution of the AGCDL. Specifically, the distribution of $T_{n}^{\mathrm{AGCDL}}$ is close to the Gumbel distribution, but the distribution of $T_{n}^{\mathrm{GCDL}}$, $T_{n}^{DL}$, and $T_{n}^{DR}$ is far from the Gumbel distribution.

## 4 Case study

We analyze a human liver cohort dataset obtained from the Sage Bionetworks synapse platform using Synapse ID syn4499. The data contain genotypes, gene expression measurements, and phenotypes of enzyme activities. Details of the dataset can be found in Gao *et al.* (2019). The phenotypes are enzyme activity measurements of cytochrome P450. The dataset consists of 170 individuals measured for 18 556 gene transcripts, 449 699 single nucleotide polymorphisms, and some covariates such as gender and age. Following Gao *et al.* (2019), we use the log-transformed activity of the *CYP2E1* gene as the response for the following analysis.

We focus on the KEGG pathway “Metabolism of Xenobiotics by Cytochrome P450” (hsa00980). There are 76 genes in this pathway, 70 of which are in our dataset. We obtain the graph structure of the 70 genes in the pathway from the KEGG pathway database using the R package *KEGGgraph*. The weight function is set as 1 if two genes are connected in the pathway. Otherwise, it is set as zero.

We apply a simple multiple testing correction method. Following Cheverud (2010) and Liu *et al.* (2016), we first calculate the effective number of tests $E_{0}$, which is given by $E_{0}=1+p^{-1}\sum_{i,j=1}^{p}\left(1-r_{ij}^{2}\right)$, where $r_{ij}$ are the pairwise correlation coefficients of the genes. The estimated $E_{0}=59.79$, which yields a gene-wide significance level of $\alpha =1-{\left(1-0.05\right)}^{1/E_{0}}=8.57\times 10^{-4}$. After obtaining *P*-values from the AGCDL, DL, or DR method, those genes with *P*-values less than α are identified as the genes affecting *CYP2E1* activity.

To select the threshold parameter *k* according to the definition of $\hat{\Theta }$, a procedure for minimizing the Frobenius norm of $\hat{\Theta }\hat{\Sigma }-I$ is adopted. Figure 10 in Web Appendix D shows that $\|\hat{\Theta }\hat{\Sigma }-I{\|}_{F}$ attains a minimum at k=10.

**Figure 6.**
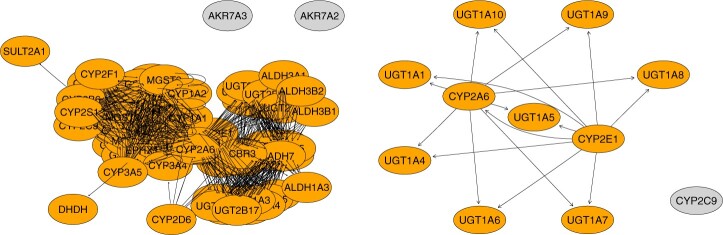
Network structure of 70 genes (left) and the genes selected by the AGCDL method and listed in Table 3 (right) from the KEGG pathway “Metabolism of Xenobiotics by Cytochrome P450”.

From Table 3, there are 11 significant genes, including *CYP2E1*. The *CYP2E1* gene has been shown to be expressed predominantly in the adult human liver (Wang *et al.* 2009). It has also been noted that the response is the CYP2E1 enzyme activity, a direct product from the expression of gene *CYP2E1*. In this analysis, the *CYP2E1* gene serves as a positive control and should show the most significant signal. Indeed, the AGCDL and DL methods identify this gene with the smallest *P*-values among its own list. However, AGCDL gives even smaller *P*-values and CI length than DL does, indicating the power of AGCDL when considering network graph information. Figure 6 shows the full KEGG connectivity information for the 70 genes and the selected gene connectivity information. Obviously, the *CYP2E1* gene has many connections with other genes. Ignoring such connectivity information could lead to low power.

**Table 3.** List of genes with *P*-value <α and CIs with the AGCDL, DL, and DR methods for pathway hsa00980. The confidence level of the CI is 1−α.

Gene	AGCDL		DL		DR	
	*P*-value	CI	*P*-value	CI	*P*-value	CI
*CYP2E1*	2.7002$\times 10^{-9}$	[0.1281, 0.4549]	1.2341$\times 10^{-6}$	[0.0957, 0.5166]	.0137	[−0.0790, 0.5263]
*UGT1A5*	8.2810$\times 10^{-5}$	[0.0374, 0.4512]	4.5124$\times 10^{-6}$	[0.0895, 0.5661]	.0199	[−0.2726, 1.5349]
*CYP2C9*	1.8722$\times 10^{-4}$	[0.0267, 0.4699]	4.5038$\times 10^{-3}$	[−0.0471, 0.5908]	.2579	[−0.3522, 0.7140]
*UGT1A8*	2.9417$\times 10^{-4}$	[0.0173, 0.4211]	3.0526$\times 10^{-1}$	[−0.2293, 0.4329]	.4146	[−0.8233, 0.4995]
*UGT1A9*	3.5224$\times 10^{-4}$	[0.0139, 0.4019]	2.2543$\times 10^{-1}$	[−0.2170, 0.4652]	.1945	[−0.4146, 0.9430]
*UGT1A10*	4.7178$\times 10^{-4}$	[0.0103, 0.4335]	5.1495$\times 10^{-2}$	[−0.0926, 0.3528]	.2788	[−1.1244, 0.5730]
*UGT1A4*	5.3260$\times 10^{-4}$	[0.0079, 0.4125]	5.5905$\times 10^{-2}$	[−0.1160, 0.4281]	.0038	[−1.7529, 0.1250]
*CYP2A6*	6.6122$\times 10^{-4}$	[0.0069, 0.6522]	2.4981$\times 10^{-3}$	[−0.0318, 0.6527]	.0577	[−0.3796, 1.3827]
*UGT1A6*	6.7374$\times 10^{-4}$	[0.0043, 0.4354]	2.4798$\times 10^{-1}$	[−0.2005, 0.4133]	.9731	[−1.0010, 1.0214]
*UGT1A7*	6.7388$\times 10^{-4}$	[0.0041, 0.4254]	4.7890$\times 10^{-3}$	[−0.0379, 0.4554]	.6788	[−1.0168, 0.7921]
*UGT1A1*	8.3379$\times 10^{-4}$	[0.0004, 0.4202]	2.6054$\times 10^{-2}$	[−0.0932, 0.4677]	.6692	[−0.8786, 1.1369]

## 5 Discussion

This paper has shown how, by incorporating auxiliary network information, the LASSO can be used to conduct inference in a high-dimensional linear regression model. The auxiliary information is presented in the form of a weight matrix *L*, which is called the graph Laplacian matrix. Also, we can use the adaptive graph Laplacian matrix (Li and Li 2010) to measure the similarity between covariates. Based on the graph Laplacian matrix, we have proposed a DL estimator, and we have shown that the asymptotic distribution of the estimator follows a normal distribution under some mild conditions. Simulation results indicate that the proposed method performs well, especially when high correlations exist between predictors.

Bühlmann (2013) proved that the DR estimator has asymptotic normality and can deal with highly correlated predictors. The simulation results show that although the DR method has higher coverage probabilities than the DL method, it has larger CIs. However, the proposed method outperforms both the DL and DR methods. This demonstrates that incorporating auxiliary network information improves accuracy.

In the node-wise regression procedure, we do not select the tuning parameter when *d* satisfies Condition (C5) in Section 2.5, and so the proposed method is competitive in terms of computational efficiency compared with the DL. In practical applications, obtaining auxiliary network information may not always be feasible. To employ the proposed method, one can initially partition the dataset into two complementary subsets. The first subset is then employed to ascertain the Laplacian matrix through a similarity metric, such as the Pearson correlation (Huang *et al.* 2011). The remaining data are then allocated for further analysis. This approach may be considered for future research work. Future work may also include bootstrapping the GCDL to gain further finite-sample improvement. The proposed method can also be extended to generalized linear models and other semi-parametric models.

## Supplementary Material

btae541_Supplementary_Data

## Data Availability

The dataset used for this study is publicly available at: https://www.synapse.org/#!Synapse:syn3275753.
