# Supplementary material for: Uncertainty quantification in high-dimensional linear models incorporating graphical structures with applications to gene set analysis
Source: Bioinformatics. 2024 Sep 10;40(9):btae541. doi: 10.1093/bioinformatics/btae541 (PMC11434165; doi:10.1093/bioinformatics/btae541)
Supplement: btae541_Supplementary_Data [file btae541_supplementary_data.pdf]

Supplementary Materials for "Uncertainty quantification in high-dimensional linear models incorporating graphical structures with applications to gene set analysis"

Xiangyong Tan[1], Xiao Zhang[2], Yuehua Cui[3] and Xu Liu[4*]

[1] *School of Statistics and Data Science, Jiangxi University of Finance and Economics, Nanchang, 330013, China*

[2] *School of Data Science, The Chinese University of Hong Kong, Shenzhen, 518172, China*

[3] *Department of Statistics and Probability, Michigan State University, East Lansing, MI 48824, U.S.A.*

[4] *School of Statistics and Management, Shanghai University of Finance and Economics, Shanghai 200433, China*

Summary

This Supporting Information file contains the proofs of Theorem 1 – 4 and related Lemmas. The simulations concerning the misspecification of the graph structure are also included.

1 Web Appendix A: Proof of main results

Lemma S.1 *Assume conditions (C1)-(C3) hold, we have*

$$\|\hat{\boldsymbol{\beta}} - \boldsymbol{\beta}\|_1 = O_p(s_0\lambda_1),$$

and

$$\frac{1}{n}\|\mathbb{X}(\hat{\boldsymbol{\beta}} - \boldsymbol{\beta})\|_2^2 = O_p(s_0\lambda_1^2).$$

Furthermore, these bounds are valid uniformly over the l_0-ball $\mathscr{B}_{l_0}(s_0) = \{\boldsymbol{\beta} : \|\boldsymbol{\beta}\|_0 \le s_0\}$.

The idea of the proof of Lemma S.1 is similar to that used in Hebiri and Van De Geer (2011).

*Corresponding author: liu.xu@sufe.edu.cn

Proof: Let $\tilde{\mathbb{X}} = (\mathbb{X}^\top, \sqrt{n\lambda_2}Q^\top)^\top$ and $\tilde{\boldsymbol{Y}} = (\boldsymbol{Y}^\top, \mathbf{0}^\top)^\top$, where $\mathbf{0}$ is a vector of size p containing only zeros. Then, the estimator $\hat{\boldsymbol{\beta}}$, which is given in (2.2), is also the minimizer of

$$\frac{1}{2n}\|\tilde{\boldsymbol{Y}} - \tilde{\mathbb{X}}\boldsymbol{\beta}\|_2^2 + \lambda_1\|\boldsymbol{\beta}\|_1.$$

Hence, by the definition of $\hat{\boldsymbol{\beta}}$, we have

$$\frac{1}{2n}\|\tilde{\boldsymbol{Y}} - \tilde{\mathbb{X}}\hat{\boldsymbol{\beta}}\|_2^2 + \lambda_1\|\hat{\boldsymbol{\beta}}\|_1 \le \frac{1}{2n}\|\tilde{\boldsymbol{Y}} - \tilde{\mathbb{X}}\boldsymbol{\beta}\|_2^2 + \lambda_1\|\boldsymbol{\beta}\|_1.$$

After some algebra, above inequality can be written as

$$\frac{1}{2n}\|\tilde{\mathbb{X}}(\hat{\boldsymbol{\beta}} - \boldsymbol{\beta})\|_2^2 \le \frac{1}{n}(\tilde{\boldsymbol{Y}} - \tilde{\mathbb{X}}\boldsymbol{\beta})^\top\tilde{\mathbb{X}}(\hat{\boldsymbol{\beta}} - \boldsymbol{\beta}) + \lambda_1\|\boldsymbol{\beta}\|_1 - \lambda_1\|\hat{\boldsymbol{\beta}}\|_1.$$

Recalling the definition of $\tilde{\mathbb{X}}$ and $\tilde{\boldsymbol{Y}}$, we can decompose following two terms

$$(\tilde{\boldsymbol{Y}} - \tilde{\mathbb{X}}\boldsymbol{\beta})^\top\tilde{\mathbb{X}}(\hat{\boldsymbol{\beta}} - \boldsymbol{\beta}) = \boldsymbol{\varepsilon}^\top\mathbb{X}(\hat{\boldsymbol{\beta}} - \boldsymbol{\beta}) - n\lambda_2\beta^\top L(\hat{\boldsymbol{\beta}} - \boldsymbol{\beta}),$$

and

$$\frac{1}{n}\|\tilde{\mathbb{X}}(\hat{\boldsymbol{\beta}} - \boldsymbol{\beta})\|_2^2 = (\hat{\boldsymbol{\beta}} - \boldsymbol{\beta})^\top K_n(\hat{\boldsymbol{\beta}} - \boldsymbol{\beta}).$$

Under condition (C2), on the event $\mathscr{E} = \{2\|\mathbb{X}^\top\boldsymbol{\varepsilon}\|_\infty/n \le \lambda_1\}$, we have

$$\begin{aligned} 0 \le& \frac{1}{n}\|\tilde{\mathbb{X}}(\hat{\boldsymbol{\beta}} - \boldsymbol{\beta})\|_2^2 \\ \le& \lambda_1\|\hat{\boldsymbol{\beta}} - \boldsymbol{\beta}\|_1 + 2\lambda_1\|\boldsymbol{\beta}\|_1 - 2\lambda_1\|\hat{\boldsymbol{\beta}}\|_1 + 2\lambda_2\|L\boldsymbol{\beta}\|_\infty\|\hat{\boldsymbol{\beta}} - \boldsymbol{\beta}\|_1 \qquad \text{(S.1)} \\ =& \frac{6}{5}\lambda_1\|\hat{\boldsymbol{\beta}} - \boldsymbol{\beta}\|_1 + 2\lambda_1\|\boldsymbol{\beta}\|_1 - 2\lambda_1\|\hat{\boldsymbol{\beta}}\|_1, \end{aligned}$$

which implies that $\frac{6}{5}\lambda_1\|\hat{\boldsymbol{\beta}} - \boldsymbol{\beta}\|_1 + 2\lambda_1\|\boldsymbol{\beta}\|_1 - 2\lambda_1\|\hat{\boldsymbol{\beta}}\|_1 \ge 0$, and further

$$\|\hat{\boldsymbol{\beta}}_{S_0^c} - \boldsymbol{\beta}_{S_0^c}\|_1 \le 4\|\hat{\boldsymbol{\beta}}_{S_0} - \boldsymbol{\beta}_{S_0}\|_1.$$

This concludes that $\hat{\boldsymbol{\beta}} - \boldsymbol{\beta}$ satisfies condition (C1).

Based on (S.1), it is easy to see that

$$\begin{aligned} &\frac{1}{n}\|\tilde{\mathbb{X}}(\hat{\boldsymbol{\beta}} - \boldsymbol{\beta})\|_2^2 + \frac{4}{5}\lambda_1\|\hat{\boldsymbol{\beta}} - \boldsymbol{\beta}\|_1 \\ &\le 2\lambda_1\|\hat{\boldsymbol{\beta}} - \boldsymbol{\beta}\|_1 + 2\lambda_1\|\boldsymbol{\beta}\|_1 - 2\lambda_1\|\hat{\boldsymbol{\beta}}\|_1 \\ &= 2\lambda_1\|\hat{\boldsymbol{\beta}}_{S_0} - \boldsymbol{\beta}_{S_0}\|_1 + 2\lambda_1\|\hat{\boldsymbol{\beta}}_{S_0^c}\|_1 + 2\lambda_1\|\boldsymbol{\beta}_{S_0}\|_1 - 2\lambda_1\|\hat{\boldsymbol{\beta}}_{S_0}\|_1 - 2\lambda_1\|\hat{\boldsymbol{\beta}}_{S_0^c}\|_1 \\ &\le 4\lambda_1\|\hat{\boldsymbol{\beta}}_{S_0} - \boldsymbol{\beta}_{S_0}\|_1 \\ &\le 4\lambda_1\sqrt{s_0}\|\hat{\boldsymbol{\beta}}_{S_0} - \boldsymbol{\beta}_{S_0}\|_2. \end{aligned}$$

The above inequality combined with condition (C1) implies that

$$\|\hat{\boldsymbol{\beta}}_{S_0} - \boldsymbol{\beta}_{S_0}\|_2 \leq 4\lambda_1\sqrt{s_0}/\phi_n,$$

and consequently

$$\|\hat{\boldsymbol{\beta}} - \boldsymbol{\beta}\|_1 \leq 20\lambda_1 s_0/\phi_n,$$

and

$$\frac{1}{n}\|\mathbb{X}(\hat{\boldsymbol{\beta}} - \boldsymbol{\beta})\|_2^2 \leq 16\lambda_1^2 s_0/\phi_n.$$

This completes the proof of Lemma S.1.

Proof of Theorem 1: It is obvious that

$$\begin{aligned} \|\hat{\Sigma}\hat{\boldsymbol{\Theta}}_j - \boldsymbol{e}_j\|_\infty =& \|\frac{1}{n}\mathbb{X}^\top\mathbb{X}\hat{\boldsymbol{\Theta}}_j - \boldsymbol{e}_j\|_\infty \\ \leq& |\frac{1}{n}\mathbb{X}_j^\top\mathbb{X}\hat{\boldsymbol{\Theta}}_j - 1| + \|\frac{1}{n}\mathbb{X}_{D_j}^\top\mathbb{X}\hat{\boldsymbol{\Theta}}_j\|_\infty + \|\frac{1}{n}\mathbb{X}_{R_j}^\top\mathbb{X}\hat{\boldsymbol{\Theta}}_j\|_\infty. \end{aligned} \tag{S.2}$$

The Karush-Kuhn-Tucker (KKT) conditions for the nodewise regression imply that

$$\mathbb{X}_{D_j}^\top(\mathbb{X}_j - \mathbb{X}_{D_j}\hat{\boldsymbol{\gamma}}_j)/n = \mathbf{0},$$

which results in that

$$\mathbb{X}_j^\top(\mathbb{X}_j - \mathbb{X}_{D_j}\hat{\boldsymbol{\gamma}}_j)/n = \|\mathbb{X}_j - \mathbb{X}_{D_j}\hat{\boldsymbol{\gamma}}_j\|_2^2/n = \hat{\tau}_j^2.$$

Thus, we have $\mathbb{X}_j^\top\mathbb{X}\hat{\boldsymbol{\Theta}}_j/n = 1$, and consequently, the first term on the right hand side in equality (S.2) is 0. By the same way, for the second term on the right hand side in equality (S.2), one gets $\mathbb{X}_{D_j}^\top\mathbb{X}\hat{\boldsymbol{\Theta}}_j/n = \mathbf{0}$.

Thus, it remains to show

$$\|\mathbb{X}_{R_j}^\top\mathbb{X}\hat{\boldsymbol{\Theta}}_j/n\|_\infty \leq \sqrt{2(n-d_j)\log(p-d_j-1)/(c\Theta_{j,j}n^2)}/\hat{\tau}_j^2$$

with probability approaching to 1 for some constant c. In fact,

$$\begin{aligned} \|\mathbb{X}_{R_j}^\top\mathbb{X}\hat{\boldsymbol{\Theta}}_j/n\|_\infty =& \|\frac{1}{n}\mathbb{X}_{R_j}^\top(\mathbb{X}_j - \mathbb{X}_{D_j}\hat{\boldsymbol{\gamma}}_j)/\hat{\tau}_j^2\|_\infty \\ =& \frac{1}{\hat{\tau}_j^2}\|\frac{1}{n}\mathbb{X}_{R_j}^\top(I - P_{D_j})\mathbb{X}_j\|_\infty, \end{aligned} \tag{S.3}$$

where $P_{D_j} = \mathbb{X}_{D_j}(\mathbb{X}_{D_j}^\top \mathbb{X}_{D_j})^{-1}\mathbb{X}_{D_j}^\top$. By some calculation, we have

$$X_{i,j}|\boldsymbol{X}_{i,D_j} \sim N(\boldsymbol{X}_{i,D_j}^\top \Theta_{D_j,j}\Theta_{j,j}^{-1}, \Theta_{j,j}^{-1}),$$
$$\boldsymbol{X}_{i,R_j}|\boldsymbol{X}_{i,D_j} \sim N(-\Theta_{R_j,R_j}^{-1}\Theta_{R_j,D_j}\boldsymbol{X}_{i,D_j}, \Theta_{R_j,R_j}^{-1}),$$

which means that

$$X_{i,j} = -\boldsymbol{X}_{i,D_j}^\top \Theta_{D_j,j}\Theta_{j,j}^{-1} + \Theta_{j,j}^{-1/2}U_{i,1}, \tag{S.4}$$
$$\boldsymbol{X}_{i,R_j} = -\Theta_{R_j,R_j}^{-1}\Theta_{R_j,D_j}\boldsymbol{X}_{i,D_j} + \Theta_{R_j,R_j}^{-1/2}\boldsymbol{U}_{i,2}. \tag{S.5}$$

where $U_{i,1} \sim N(0,1)$, $\boldsymbol{U}_{i,2} \sim N(\boldsymbol{0}, I_{r_j,r_j})$ and $U_{i,1}$ is independent of $\boldsymbol{U}_{i,2}$.

Let $\boldsymbol{U}_1 = (U_{1,1},\dots,U_{n,1})^\top$ and $U_2 \equiv (\boldsymbol{U}_2^{(1)},\dots,\boldsymbol{U}_2^{(r_j)}) = (\boldsymbol{U}_{1,2},\dots,\boldsymbol{U}_{n,2})^\top$. Then, by (S.4) and (S.5), $\|\mathbb{X}_{R_j}^\top\left(I - P_{D_j}\right)\mathbb{X}_j\|_\infty$ can be rewritten as

$$\begin{aligned}
&\|\mathbb{X}_{R_j}^\top\left(I - P_{D_j}\right)\mathbb{X}_j\|_\infty \\
&= \|\left(\Theta_{R_j,R_j}^{-1/2}U_2^\top - \Theta_{R_j,R_j}^{-1}\Theta_{R_j,D_j}\mathbb{X}_{D_j}^\top\right)\left(I - P_{D_j}\right)\left(\Theta_{j,j}^{-1/2}\boldsymbol{U}_1 - \mathbb{X}_{D_j}\Theta_{D_j,j}\Theta_{j,j}^{-1}\right)\|_\infty \\
&= \|\Theta_{R_j,R_j}^{-1/2}U_2^\top\left(I - P_{D_j}\right)\Theta_{j,j}^{-1/2}\boldsymbol{U}_1\|_\infty \\
&\le c\|U_2^\top\left(I - P_{D_j}\right)\Theta_{j,j}^{-1/2}\boldsymbol{U}_1\|_\infty.
\end{aligned}$$

Thus, it suffices to show

$$\|U_2^\top\left(I - P_{D_j}\right)\Theta_{j,j}^{-1/2}\boldsymbol{U}_1/n\|_\infty \le \sqrt{2(n-d_j)\log(p-d_j-1)/(c\Theta_{j,j}n^2)}$$

with probability tending to 1 for some constant c.

Let $A^{(j)} = I - P_{D_j}$. Then $A^{(j)}$ can be expressed as $A^{(j)} = \sum_{i=1}^n s_i u_i u_i^\top$, where s_i is the singular value of $A^{(j)}$, u_i is the eigenvector corresponding to the eigenvalue s_i. Then, for the kth row of $U_2^\top\left(I - P_{D_j}\right)\Theta_{j,j}^{-1/2}\boldsymbol{U}_1$, by Bernstein-type inequality for centered subexponential random variables (Proposition 5.16 in Vershynin (2010)), we have

$$\begin{aligned}
&P\left(|\frac{1}{n}(\boldsymbol{U}_2^{(k)})^\top\left(I - P_{D_j}\right)\Theta_{j,j}^{-1/2}\boldsymbol{U}_1| \ge t\Big|X_{D_j}\right) \\
&= P\left(|\sum_{i=1}^n s_i\langle u_i, \boldsymbol{U}_2^{(k)}\rangle\langle u_i, \boldsymbol{U}_1\rangle| \ge \Theta_{j,j}^{1/2}nt\Big|X_{D_j}\right) \qquad \text{(S.6)} \\
&\le 2\exp\left(-c\frac{\Theta_{j,j}n^2t^2}{n-d_j}\right),
\end{aligned}$$

where $\boldsymbol{U}_2^{(k)}$ is the kth column of $\boldsymbol{U}_2$. Taking $t = \sqrt{2(n-d_j)\log(p-d_j-1)/(c\Theta_{j,j}n^2)}$, (S.6) implies that

$$\begin{aligned}
&P\left(\|\frac{1}{n}\mathbb{X}_{R_j}^\top(I-P_{D_j})\mathbb{X}_j\|_\infty \geq t\right)\\
&\leq (p-d_j-1)E\left(P\left(|\frac{1}{n}\mathbb{X}_k^\top(I-P_{D_j})\mathbb{X}_j| \geq t \mid \mathbb{X}_{D_j}\right)\right) \qquad \text{(S.7)}\\
&\leq 2(p-d_j-1)^{-1}.
\end{aligned}$$

Thus, we complete the proof by combining (S.7) and (S.3).

Lemma S.2 *Assume conditions (C4), (C6) hold, for any $j \in \{1,\ldots,p\}$, we have*

$$\|\hat{\boldsymbol{\gamma}}_j - \boldsymbol{\gamma}_j\|_1 = O_p(d\sqrt{\log d/n}), \qquad \text{(S.8)}$$

$$|\hat{\tau}_j^2 - \tau_j^2| = O_p(\sqrt{d\log d/n}), \qquad \text{(S.9)}$$

$$\left|\frac{1}{\hat{\tau}_j^2} - \frac{1}{\tau_j^2}\right| = O_p(\sqrt{d\log d/n}). \qquad \text{(S.10)}$$

Proof: Fist, we are going to show that

$$\|\boldsymbol{\gamma}_j\|_1 = O(\sqrt{d}), \qquad \text{(S.11)}$$

Let Σ_{D_j} denote the submatrix of Σ consisting only of the rows and columns indexed by D_j. Note that

$$\frac{\boldsymbol{\gamma}_j^\top\Sigma_{D_j}\boldsymbol{\gamma}_j}{\boldsymbol{\gamma}_j^\top\boldsymbol{\gamma}_j} \geq \Lambda_{\min}(\Sigma_{D_j}) \geq \Lambda_{\min}(\Sigma),$$

that is

$$\frac{\boldsymbol{\gamma}_j^\top\Sigma_{D_j}\boldsymbol{\gamma}_j}{\Lambda_{\min}(\Sigma)} \geq \boldsymbol{\gamma}_j^\top\boldsymbol{\gamma}_j.$$

Since $\boldsymbol{\eta}_j = \mathbb{X}_j - \mathbb{X}_{D_j}\boldsymbol{\gamma}_j$, we have $E(X_{1,j}^2) = \boldsymbol{\gamma}_j^\top\Sigma_{D_j}\boldsymbol{\gamma}_j + E(\eta_{1,j}^2) = 1$. Hence $\boldsymbol{\gamma}_j^\top\boldsymbol{\gamma}_j \leq 1/\Lambda_{\min}(\Sigma)$, which implies $\|\boldsymbol{\gamma}_j\|_1 = O(\sqrt{d_j}) = O(\sqrt{d})$.

Next we are going to show

$$\|\mathbb{X}_{D_j}^\top\boldsymbol{\eta}_j/n\|_\infty = O_p(\sqrt{\log d/n}). \qquad \text{(S.12)}$$

Let $\kappa_{1,D_j} = \max\{\|X_{i,j}\eta_{i,j}\|_{\psi_1}, j \in D_j\}$. By Bernstein-type inequality for centered subexponential random variables (Proposition 5.16 in Vershynin (2010)), we have

$$\begin{aligned}
P\left(\|\mathbb{X}_{D_j}^\top\boldsymbol{\eta}_j/n\|_\infty > t\right) =& P\left(\max_{j\in D_j}|\mathbb{X}_j^\top\boldsymbol{\eta}_j| > nt\right)\\
\leq& 2\sum_{j\in D_j}\exp\left[-c\min\left(\frac{n^2t^2}{n\kappa_{1,D_j}^2}, \frac{nt}{\kappa_{1,D_j}}\right)\right].
\end{aligned}$$

Taking $t = \sqrt{2\log(d_j)\kappa_{1,D_j}^2/(cn)}$, we have $P\left(\|\mathbb{X}_{D_j}^\top \boldsymbol{\eta}_j/n\|_\infty > t\right) \le 2d_j^{-1}$, which implies

$$\|\mathbb{X}_{D_j}^\top \boldsymbol{\eta}_j/n\|_\infty = O_p(\sqrt{\log d_j/n}) = O_p(\sqrt{\log d/n}).$$

Now we focus on the proof of (S.8) - (S.10). We first consider $\|\hat{\boldsymbol{\gamma}}_j - \boldsymbol{\gamma}_j\|_1$. Let $\tilde{\lambda} \asymp \sqrt{\log d/n}$ and $\mathscr{E}_j = \{2\|\mathbb{X}_{D_j}^\top \boldsymbol{\eta}_j\|_\infty/n \le \tilde{\lambda}\}$. It is easy to see that (S.12) implies $P(\mathscr{E}_j) \to 1$. By the definition of $\hat{\boldsymbol{\gamma}}_j$, we have, on the event $\mathscr{E}_j$,

$$\frac{1}{n}\|\mathbb{X}_j - \mathbb{X}_{D_j}\hat{\boldsymbol{\gamma}}_j\|_2^2 \le \frac{1}{n}\|\mathbb{X}_j - \mathbb{X}_{D_j}\boldsymbol{\gamma}_j\|_2^2 = \frac{1}{n}\|\boldsymbol{\eta}_j\|_2^2,$$

which implies that

$$\begin{aligned}\frac{1}{n}\|\mathbb{X}_{D_j}(\hat{\boldsymbol{\gamma}}_j - \boldsymbol{\gamma}_j)\|_2^2 \le& \frac{2}{n}(\hat{\boldsymbol{\gamma}}_j - \boldsymbol{\gamma}_j)^\top \mathbb{X}_{D_j}^\top \boldsymbol{\eta}_j \\ \le& \|\hat{\boldsymbol{\gamma}}_j - \boldsymbol{\gamma}_j\|_1 \|2\mathbb{X}_{D_j}^\top \boldsymbol{\eta}_j/n\|_\infty \\ \le& \tilde{\lambda}\|\hat{\boldsymbol{\gamma}}_j - \boldsymbol{\gamma}_j\|_1.\end{aligned}$$

By condition (C6) combining with Cauchy-Schwarz inequality, we have

$$\begin{aligned}\frac{1}{n}\|\mathbb{X}_{D_j}(\hat{\boldsymbol{\gamma}}_j - \boldsymbol{\gamma}_j)\|_2^2 \ge& \Lambda_{\min}(\frac{1}{n}\mathbb{X}_{D_j}^\top \mathbb{X}_{D_j})\|\hat{\boldsymbol{\gamma}}_j - \boldsymbol{\gamma}_j\|_2^2 \\ \ge& \Lambda_{\min}(\frac{1}{n}\mathbb{X}_{D_j}^\top \mathbb{X}_{D_j})\|\hat{\boldsymbol{\gamma}}_j - \boldsymbol{\gamma}_j\|_1^2/d_j.\end{aligned}$$

Hence, $\Lambda_{\min}(\frac{1}{n}\mathbb{X}_{D_j}^\top \mathbb{X}_{D_j})\|\hat{\boldsymbol{\gamma}}_j - \boldsymbol{\gamma}_j\|_1^2/d_j \le \tilde{\lambda}\|\hat{\boldsymbol{\gamma}}_j - \boldsymbol{\gamma}_j\|_1$, which means

$$\|\hat{\boldsymbol{\gamma}}_j - \boldsymbol{\gamma}_j\|_1 \le \tilde{\lambda} d_j/\Lambda_{\min}(\frac{1}{n}\mathbb{X}_{D_j}^\top \mathbb{X}_{D_j}).$$

Then, we have $\|\hat{\boldsymbol{\gamma}}_j - \boldsymbol{\gamma}_j\|_1 = O_p(d\sqrt{\log d/n})$.

Next we consider $|\hat{\tau}_j^2 - \tau_j^2|$. By the definition of $\hat{\tau}_j^2$ and $\mathbb{X}_j = \mathbb{X}_{D_j}\boldsymbol{\gamma}_j + \boldsymbol{\eta}_j$,

$$\begin{aligned}\hat{\tau}_j^2 =& \frac{1}{n}\left(\mathbb{X}_j - \mathbb{X}_{D_j}\hat{\boldsymbol{\gamma}}_j\right)^\top \boldsymbol{X}_j \\ =& \frac{1}{n}\left(\boldsymbol{\eta}_j - \mathbb{X}_{D_j}(\hat{\boldsymbol{\gamma}}_j - \boldsymbol{\gamma}_j)\right)^\top \left(\mathbb{X}_{D_j}\boldsymbol{\gamma}_j + \boldsymbol{\eta}_j\right) \\ =& \frac{1}{n}\boldsymbol{\eta}_j^\top \boldsymbol{\eta}_j + \frac{1}{n}\boldsymbol{\eta}_j^\top \mathbb{X}_{D_j}\boldsymbol{\gamma}_j - \frac{1}{n}\left(\hat{\boldsymbol{\gamma}}_j - \boldsymbol{\gamma}_j\right)^\top \mathbb{X}_{D_j}^\top \mathbb{X}_{D_j}\boldsymbol{\gamma}_j - \frac{1}{n}\left(\hat{\boldsymbol{\gamma}}_j - \boldsymbol{\gamma}_j\right)^\top \mathbb{X}_{D_j}^\top \boldsymbol{\eta}_j.\end{aligned}$$

It follows from the above equation that

$$\begin{aligned}|\hat{\tau}_j^2 - \tau_j^2| \le& \left|\boldsymbol{\eta}_j^\top \boldsymbol{\eta}_j/n - \tau_j^2\right| + \left|\boldsymbol{\eta}_j^\top \mathbb{X}_{D_j}\boldsymbol{\gamma}_j/n\right| \\ &+ \left|\left(\hat{\boldsymbol{\gamma}}_j - \boldsymbol{\gamma}_j\right)^\top \mathbb{X}_{D_j}^\top \boldsymbol{\eta}_j/n\right| + \left|\left(\hat{\boldsymbol{\gamma}}_j - \boldsymbol{\gamma}_j\right)^\top \mathbb{X}_{D_j}^\top \mathbb{X}_{D_j}\boldsymbol{\gamma}_j/n\right|.\end{aligned} \tag{S.13}$$

Since $\boldsymbol{\eta}_j^\top \boldsymbol{\eta}_j/n - \tau_j^2 = \frac{1}{n}\sum_{i=1}^n \left(\eta_{i,j}^2 - E(\eta_{i,j}^2)\right)$ is a sum of i.i.d. sub-exponential variables with mean zero. Let $M > 0$ and $\kappa_j = \|\eta_{i,j}^2 - E(\eta_{i,j}^2)\|_{\psi_1}$. By Bernstein-type inequality for centered subexponential random variables (Proposition 5.16 in Vershynin (2010)), we have

$$\begin{aligned}
P\left(\left|\boldsymbol{\eta}_j^\top \boldsymbol{\eta}_j/n - \tau_j^2\right| > Mn^{-1/2}\right) =& P\left(\left|\sum_{i=1}^n \left(\eta_{i,j}^2 - E\left(\eta_{i,j}^2\right)\right)\right| > Mnn^{-1/2}\right) \\
\leq& 2\exp\left[-c\min\left(\frac{M^2 n}{n\kappa_j^2}, \frac{Mn^{1/2}}{\kappa_j}\right)\right] \\
=& 2\exp\left(-cM^2/\kappa_j^2\right),
\end{aligned}$$

which implies that $\left|\boldsymbol{\eta}_j^\top \boldsymbol{\eta}_j/n - \tau_j^2\right| = O_p(n^{-1/2})$.

By (S.11) and (S.12), the second term on the right hand side in inequality (S.13) is

$$\left|\boldsymbol{\eta}_j^\top \mathbb{X}_{D_j}\boldsymbol{\gamma}_j/n\right| \leq \|\boldsymbol{\gamma}_j\|_1 \|\mathbb{X}_{D_j}^\top \boldsymbol{\eta}_j/n\|_\infty = O_p(\sqrt{d_j}\sqrt{\log d_j/n}) = O_p(\sqrt{d\log d/n}).$$

For the third term on the right hand side in inequality (S.13), by (S.12), we have

$$\left|\left(\hat{\boldsymbol{\gamma}}_j - \boldsymbol{\gamma}_j\right)^\top \mathbb{X}_{D_j}^\top \boldsymbol{\eta}_j/n\right| \leq \|\hat{\boldsymbol{\gamma}}_j - \boldsymbol{\gamma}_j\|_1 \|\mathbb{X}_{D_j}^\top \boldsymbol{\eta}_j/n\|_\infty = O_p(d\log d/n).$$

It remains to show $\left|\left(\hat{\boldsymbol{\gamma}}_j - \boldsymbol{\gamma}_j\right)^\top \mathbb{X}_{D_j}^\top \mathbb{X}_{D_j}\boldsymbol{\gamma}_j/n\right| = O_p(\sqrt{d\log d/n})$. By the KKT conditions for the node-wise regression one gets

$$\frac{1}{n}\mathbb{X}_{D_j}^\top(\mathbb{X}_j - \mathbb{X}_{D_j}\hat{\boldsymbol{\gamma}}_j) = 0,$$

which, by using $\boldsymbol{\eta}_j = \mathbb{X}_j - \mathbb{X}_{D_j}\boldsymbol{\gamma}_j$, is equivalent to

$$\frac{1}{n}\mathbb{X}_{D_j}^\top \mathbb{X}_{D_j}(\hat{\boldsymbol{\gamma}}_j - \boldsymbol{\gamma}_j) = \frac{1}{n}\mathbb{X}_{D_j}^\top \boldsymbol{\eta}_j.$$

Then, by (S.11) and (S.12), we conclude that

$$\begin{aligned}
\left|\left(\hat{\boldsymbol{\gamma}}_j - \boldsymbol{\gamma}_j\right)^\top \mathbb{X}_{D_j}^\top \mathbb{X}_{D_j}\boldsymbol{\gamma}_j/n\right| \leq& \|\boldsymbol{\gamma}_j\|_1 \|\mathbb{X}_{D_j}^\top \boldsymbol{\eta}_j/n\|_\infty \\
=& O_p(\sqrt{d\log d/n}).
\end{aligned}$$

Thus, $\max_{1\leq j\leq p} |\hat{\tau}_j^2 - \tau_j^2| = O_p(\sqrt{d\log d/n})$.

Finally, note that $\tau_j^2 = 1/\Theta_{j,j} \geq \Lambda_{\min}(\Sigma)$ for all $j = 1, \ldots, p$. Then, by condition (C4), we have $\min_{1\leq j\leq p} \tau_j^2$ is bounded away from zero, and so

$$\min_{1\leq j\leq p} \hat{\tau}_j^2 = \min_{1\leq j\leq p} (\hat{\tau}_j^2 - \tau_j^2 + \tau_j^2) \geq \min_{1\leq j\leq p} \tau_j^2 - \max_{1\leq j\leq p} |\hat{\tau}_j^2 - \tau_j^2|,$$

is bounded away from zero with probability tending to one. This implies

$$\left|\frac{1}{\hat{\tau}_j^2}-\frac{1}{\tau_j^2}\right|=\left|\frac{\hat{\tau}_j^2-\tau_j^2}{\hat{\tau}_j^2\tau_j^2}\right|=O_p(\sqrt{d\log d/n}).$$

Lemma S.3 *Assume conditions in Lemma S.2 hold. Then, for any $j\in\{1,\ldots,p\}$, we have*

$$\|\hat{\boldsymbol{\Theta}}_j-\boldsymbol{\Theta}_j\|_1=O_p(d\sqrt{\log d/n}).$$

Proof: Using (S.9), (S.10) and (S.11), obviously we have

$$\begin{aligned}\|\hat{\boldsymbol{\Theta}}_j-\boldsymbol{\Theta}_j\|_1 \le&\left|\frac{1}{\hat{\tau}_j^2}-\frac{1}{\tau_j^2}\right|+\left\|\frac{\hat{\boldsymbol{\gamma}}_j}{\hat{\tau}_j^2}-\frac{\boldsymbol{\gamma}_j}{\tau_j^2}\right\|_1\\ =&\left|\frac{1}{\hat{\tau}_j^2}-\frac{1}{\tau_j^2}\right|+\left\|\frac{\hat{\boldsymbol{\gamma}}_j}{\hat{\tau}_j^2}-\frac{\boldsymbol{\gamma}_j}{\hat{\tau}_j^2}+\frac{\boldsymbol{\gamma}_j}{\hat{\tau}_j^2}-\frac{\boldsymbol{\gamma}_j}{\tau_j^2}\right\|_1\\ \le&\left|\frac{1}{\hat{\tau}_j^2}-\frac{1}{\tau_j^2}\right|+\frac{1}{\hat{\tau}_j^2}\|\hat{\boldsymbol{\gamma}}_j-\boldsymbol{\gamma}_j\|_1+\|\boldsymbol{\gamma}_j\|_1\left|\frac{1}{\hat{\tau}_j^2}-\frac{1}{\tau_j^2}\right|\\ =&O_p(d\sqrt{\log d/n}).\end{aligned}$$

This completes the proof of this Lemma.

Proof of Theorem 2:

By Lemma S.1, Lemma S.3, Theorem 1, conditions (C3) and (C5) and $\hat{\tau}_j^2=O_p(1)$, it is straightforward to see that

$$|\sqrt{n}(\hat{\Sigma}\hat{\boldsymbol{\Theta}}_j-\boldsymbol{e}_j)^\top(\hat{\boldsymbol{\beta}}-\boldsymbol{\beta})|\le\sqrt{n}\|\hat{\Sigma}\hat{\boldsymbol{\Theta}}_j-\boldsymbol{e}_j\|_\infty\|\hat{\boldsymbol{\beta}}-\boldsymbol{\beta}\|_1=o_p(1),$$

and

$$|\sqrt{n}(\hat{\boldsymbol{\Theta}}_j-\boldsymbol{\Theta}_j)^\top\mathbb{X}^\top\boldsymbol{\varepsilon}/n|\le\sqrt{n}\|\hat{\boldsymbol{\Theta}}_j-\boldsymbol{\Theta}_j\|_1\|\mathbb{X}^\top\boldsymbol{\varepsilon}/n\|_\infty=o_p(1).$$

Thus, by (2.5) and the above two equalities imply that

$$\sqrt{n}(\hat{b}_j-\beta_j)\xrightarrow{\mathcal{L}}\left(0,\sigma^2\boldsymbol{\Theta}_j^\top\Sigma\boldsymbol{\Theta}_j\right).$$

What remains to be shown is that $\left|\hat{\boldsymbol{\Theta}}_j^\top\hat{\Sigma}\hat{\boldsymbol{\Theta}}_j-\boldsymbol{\Theta}_j^\top\Sigma\boldsymbol{\Theta}_j\right|=o_p(1)$. By (S.10), (S.11) and Theorem 1, we have

$$\begin{aligned}&\left|\hat{\boldsymbol{\Theta}}_j^\top\hat{\Sigma}\hat{\boldsymbol{\Theta}}_j-\boldsymbol{\Theta}_j^\top\Sigma\boldsymbol{\Theta}_j\right|\\ =&\left|\left(\hat{\Sigma}\hat{\boldsymbol{\Theta}}_j-\boldsymbol{e}_j\right)^\top\hat{\boldsymbol{\Theta}}_j+\hat{\Theta}_{j,j}-\Theta_{j,j}\right|\\ \le&\|\hat{\Sigma}\hat{\boldsymbol{\Theta}}_j-\boldsymbol{e}_j\|_\infty\|\hat{\Theta}_j\|_1+\left|\hat{\Theta}_{j,j}-\Theta_{j,j}\right|\\ =&\|\hat{\Sigma}\hat{\boldsymbol{\Theta}}_j-\boldsymbol{e}_j\|_\infty\|\hat{\boldsymbol{\Theta}}_j\|_1+\left|\frac{1}{\hat{\tau}_j^2}-\frac{1}{\tau_j^2}\right|=o_p(1).\end{aligned}$$

This completes the proof of Theorem 2.

Proof of Theorem 3:

The idea of the proof of Theorem 3 is similar to that used in Caner and Kock (2018).

Let $\Delta_j = \sqrt{n}(\hat{\Sigma}\hat{\boldsymbol{\Theta}}_j - \boldsymbol{e}_j)^\top(\hat{\boldsymbol{\beta}} - \boldsymbol{\beta})$. For any $\epsilon > 0$, define events

$$A_1 = \{\sup_{\beta \in \mathscr{B}_{l_0}(s_0)} |\Delta_j| < \epsilon\}, \quad A_2 = \{|(\hat{\boldsymbol{\Theta}}_j - \boldsymbol{\Theta}_j)^\top \mathbb{X}^\top \boldsymbol{\varepsilon}/\sqrt{n}| < \epsilon\},$$

and

$$A_3 = \left\{ \sup_{\beta \in \mathscr{B}_{l_0}(s_0)} \left| \frac{\sqrt{\hat{\sigma}^2 \hat{\boldsymbol{\Theta}}_j^\top \hat{\Sigma} \hat{\boldsymbol{\Theta}}_j}}{\sqrt{\sigma^2 \boldsymbol{\Theta}_j^T \Sigma \boldsymbol{\Theta}_j}} - 1 \right| < \epsilon \right\}.$$

By Lemma (S.1), Theorem 2 and $\boldsymbol{\Theta}_j^\top \Sigma \boldsymbol{\Theta}_j$ being bounded away from zero, the probabilities of these three events all tend to one. Thus, for every $t \in R$,

$$\begin{aligned}
&\left| P\left(\frac{\sqrt{n}(\hat{b}_j - \beta_j)}{\sqrt{\hat{\sigma}^2 \hat{\boldsymbol{\Theta}}_j^\top \hat{\Sigma} \hat{\boldsymbol{\Theta}}_j}} \le t \right) - \Phi(t) \right| \\
&= \left| P\left(\frac{\hat{\boldsymbol{\Theta}}_j^\top \mathbb{X}^\top \boldsymbol{\varepsilon}/\sqrt{n}}{\sqrt{\hat{\sigma}^2 \hat{\boldsymbol{\Theta}}_j^\top \hat{\Sigma} \hat{\boldsymbol{\Theta}}_j}} - \frac{\Delta_j}{\sqrt{\hat{\sigma}^2 \hat{\boldsymbol{\Theta}}_j^\top \hat{\Sigma} \hat{\boldsymbol{\Theta}}_j}} \le t \right) - \Phi(t) \right| \qquad \text{(S.14)} \\
&\le \left| P\left(\frac{\hat{\Theta}_j^\top \mathbb{X}^\top \boldsymbol{\varepsilon}/\sqrt{n}}{\sqrt{\hat{\sigma}^2 \hat{\boldsymbol{\Theta}}_j^\top \hat{\Sigma} \hat{\boldsymbol{\Theta}}_j}} - \frac{\Delta_j}{\sqrt{\hat{\sigma}^2 \hat{\boldsymbol{\Theta}}_j^\top \hat{\Sigma} \hat{\boldsymbol{\Theta}}_j}} \le t, \cap_{j=1}^3 A_j \right) - \Phi(t) \right| + P(\cup_{j=1}^3 A_j^c)
\end{aligned}$$

Because $\boldsymbol{\Theta}_j^\top \Sigma \boldsymbol{\Theta}_j$ dose not depend on $\boldsymbol{\beta}$ and is bounded away from zero, there exists a positive constant C such that

$$\begin{aligned}
&P\left(\frac{\hat{\boldsymbol{\Theta}}_j^\top \mathbb{X}^\top \boldsymbol{\varepsilon}/\sqrt{n}}{\sqrt{\hat{\sigma}^2 \hat{\boldsymbol{\Theta}}_j^\top \hat{\Sigma} \hat{\boldsymbol{\Theta}}_j}} - \frac{\Delta_j}{\sqrt{\hat{\sigma}^2 \hat{\boldsymbol{\Theta}}_j^\top \hat{\Sigma} \hat{\boldsymbol{\Theta}}_j}} \le t, \cap_{j=1}^3 A_j \right) \\
&= P\left(\frac{\hat{\boldsymbol{\Theta}}_j^\top \mathbb{X}^\top \boldsymbol{\varepsilon}/\sqrt{n}}{\sqrt{\sigma^2 \boldsymbol{\Theta}_j^\top \Sigma \boldsymbol{\Theta}_j}} - \frac{\Delta_j}{\sqrt{\sigma^2 \boldsymbol{\Theta}_j^\top \Sigma \boldsymbol{\Theta}_j}} \le t \frac{\sqrt{\hat{\sigma}^2 \hat{\boldsymbol{\Theta}}_j^\top \hat{\Sigma} \hat{\boldsymbol{\Theta}}_j}}{\sqrt{\sigma^2 \boldsymbol{\Theta}_j^\top \Sigma \boldsymbol{\Theta}_j}}, \cap_{j=1}^3 A_j \right) \\
&\le P\left(\frac{\boldsymbol{\Theta}_j^\top \mathbb{X}^\top \boldsymbol{\varepsilon}/\sqrt{n}}{\sqrt{\sigma^2 \boldsymbol{\Theta}_j^\top \Sigma \boldsymbol{\Theta}_j}} \le t(1+\epsilon) + \frac{2\epsilon}{\sqrt{\sigma^2 \boldsymbol{\Theta}_j^\top \Sigma \boldsymbol{\Theta}_j}} \right) \\
&\le P\left(\frac{\boldsymbol{\Theta}_j^\top \mathbb{X}^\top \boldsymbol{\varepsilon}/\sqrt{n}}{\sqrt{\sigma^2 \boldsymbol{\Theta}_j^\top \Sigma \boldsymbol{\Theta}_j}} \le t(1+\epsilon) + 2C\epsilon \right).
\end{aligned}$$

As the right hand side in the above display does not dependent on $\boldsymbol{\beta}$ and with the asymptotic normality of $\boldsymbol{\Theta}_j^\top \mathbb{X}^\top \boldsymbol{\varepsilon}/\sqrt{n}/\sqrt{\sigma^2 \boldsymbol{\Theta}_j^\top \Sigma \boldsymbol{\Theta}_j}$, for n sufficiently large, we have

$$\begin{aligned}
&\sup_{\beta \in \mathscr{B}_{l_0}(s_0)} P\left(\frac{\hat{\boldsymbol{\Theta}}_j^\top \mathbb{X}^\top \boldsymbol{\varepsilon}/\sqrt{n}}{\sqrt{\hat{\sigma}^2 \hat{\boldsymbol{\Theta}}_j^\top \hat{\Sigma} \hat{\boldsymbol{\Theta}}_j}} - \frac{\Delta_j}{\sqrt{\hat{\sigma}^2 \hat{\boldsymbol{\Theta}}_j^\top \hat{\Sigma} \hat{\boldsymbol{\Theta}}_j}} \le t, \cap_{j=1}^3 A_j \right) \\
&\le P\left(\frac{\boldsymbol{\Theta}_j^\top \mathbb{X}^\top \boldsymbol{\varepsilon}/\sqrt{n}}{\sqrt{\sigma^2 \boldsymbol{\Theta}_j^\top \Sigma \boldsymbol{\Theta}_j}} \le t(1+\epsilon) + 2C\epsilon \right) \\
&\le \Phi(t(1+\epsilon) + 2C\epsilon)) + \epsilon.
\end{aligned} \tag{S.15}$$

Similarity, as $P(\cap_{j=1}^3 A_j)$ can be made arbitrarily close to one, we can conclude that

$$\begin{aligned}
&\inf_{\beta \in \mathscr{B}_{l_0}(s_0)} P\left(\frac{\hat{\boldsymbol{\Theta}}_j^\top \mathbb{X}^\top \boldsymbol{\varepsilon}/\sqrt{n}}{\sqrt{\hat{\sigma}^2 \hat{\boldsymbol{\Theta}}_j^\top \hat{\Sigma} \hat{\boldsymbol{\Theta}}_j}} - \frac{\Delta_j}{\sqrt{\hat{\sigma}^2 \hat{\boldsymbol{\Theta}}_j^\top \hat{\Sigma} \hat{\boldsymbol{\Theta}}_j}} \le t, \cap_{j=1}^3 A_j \right) \\
&\ge P\left(\frac{\boldsymbol{\Theta}_j^\top \mathbb{X}^\top \boldsymbol{\varepsilon}/\sqrt{n}}{\sqrt{\sigma^2 \boldsymbol{\Theta}_j^\top \Sigma \boldsymbol{\Theta}_j}} \le t(1-\epsilon) - 2C\epsilon, \cap_{j=1}^3 A_j \right) \\
&\ge P\left(\frac{\boldsymbol{\Theta}_j^\top \mathbb{X}^\top \boldsymbol{\varepsilon}/\sqrt{n}}{\sqrt{\sigma^2 \boldsymbol{\Theta}_j^\top \Sigma \boldsymbol{\Theta}_j}} \le t(1-\epsilon) - 2C\epsilon \right) + P(\cap_{j=1}^3 A_j) - 1 \\
&\ge \Phi(t(1+\epsilon) - 2C\epsilon)) - 2\epsilon.
\end{aligned} \tag{S.16}$$

By (S.14), (S.15) and (S.16) and $P(\cup_{j=1}^3 A_j^c) \to 0$, we have

$$\sup_{\beta \in \mathscr{B}_{l_0}(s_0)} \left| P\left(\frac{\sqrt{n}(\hat{b}_j - \beta_j)}{\sqrt{\hat{\sigma}^2 \hat{\boldsymbol{\Theta}}_j^\top \hat{\Sigma} \hat{\boldsymbol{\Theta}}_j}} \le t \right) - \Phi(t) \right| \to 0. \tag{S.17}$$

By (S.17) and the fact that $\Phi(t)$ is continuous (for more details, please see the proof of Lemma 2 in Mikusheva (2007)), we have

$$\sup_{t \in R} \sup_{\beta \in \mathscr{B}_{l_0}(s_0)} \left| P\left(\frac{\sqrt{n}(\hat{b}_j - \beta_j)}{\sqrt{\hat{\sigma}^2 \hat{\boldsymbol{\Theta}}_j^\top \hat{\Sigma} \hat{\boldsymbol{\Theta}}_j}} \le t \right) - \Phi(t) \right| \to 0.$$

The remaining conclusions of the Theorem 3 can be easily obtained from the above equality and are omitted here. Thus, we complete the proof of Theorem 3.

Lemma S.4 *Define* $\tilde{T}_n = \max_{1 \le j \le p} \tilde{T}_j^2$, $\check{T}_n = \max_{1 \le j \le p} \check{T}_j^2$, *where*

$$\tilde{T}_j = \frac{\hat{\boldsymbol{\Theta}}_j^\top \mathbb{X}^\top \boldsymbol{\varepsilon}}{\sqrt{n \hat{\sigma}^2 \hat{\boldsymbol{\Theta}}_j^\top \hat{\Sigma} \hat{\boldsymbol{\Theta}}_j}}, \quad \textit{and}, \quad \check{T}_j = \frac{\boldsymbol{\Theta}_j^\top \mathbb{X}^\top \boldsymbol{\varepsilon}}{\sqrt{n \sigma^2 \boldsymbol{\Theta}_j^\top \Sigma \boldsymbol{\Theta}_j}}, j = 1, \ldots, p.$$

Under the conditions of Theorem 5, the following events

$$B_1 = \left\{|\tilde{T}_n - T_n| = o(1)\right\}, \quad \textit{and} \quad B_2 = \left\{|\tilde{T}_n - \check{T}_n| = o(1)\right\}$$

hold with probability at least $1 - O(p^{-c})$ *for some constant* $c > 0$.

Proof: For event B_2, we first claim that

$$\max_{1\le j\le p} |\tilde{T}_j - \check{T}_j| = o_p(1/\sqrt{\log p}). \tag{S.18}$$

In fact, for any $j \in \{1, \dots, p\}$, we have

$$\begin{aligned}
&|\tilde{T}_j - \check{T}_j| \\
&= \Big|\frac{\hat{\mathbf{\Theta}}_j^\top \mathbb{X}^\top \boldsymbol{\varepsilon}}{\sqrt{n\hat{\sigma}^2 \hat{\mathbf{\Theta}}_j^\top \hat{\Sigma}\hat{\mathbf{\Theta}}_j}} - \frac{\Theta_j^\top \mathbb{X}^\top \boldsymbol{\varepsilon}}{\sqrt{n\sigma^2\Theta_{j,j}}}\Big| \\
&\le \Big|\frac{\hat{\mathbf{\Theta}}_j^\top \mathbb{X}^\top \boldsymbol{\varepsilon}}{\sqrt{n\hat{\sigma}^2 \hat{\mathbf{\Theta}}_j^\top \hat{\Sigma}\hat{\mathbf{\Theta}}_j}} - \frac{\hat{\mathbf{\Theta}}_j^\top \mathbb{X}^\top \boldsymbol{\varepsilon}}{\sqrt{n\sigma^2\Theta_{j,j}}}\Big| + \Big|\frac{\hat{\mathbf{\Theta}}_j^\top \mathbb{X}^\top \boldsymbol{\varepsilon}}{\sqrt{n\sigma^2\Theta_{j,j}}} - \frac{\mathbf{\Theta}_j^\top \mathbb{X}^\top \boldsymbol{\varepsilon}}{\sqrt{n\sigma^2\Theta_{j,j}}}\Big| \\
&= I_1 + I_2.
\end{aligned} \tag{S.19}$$

For I_1, we have

$$\begin{aligned}
I_1 =& \Big|\frac{\hat{\mathbf{\Theta}}_j^\top \mathbb{X}^\top \boldsymbol{\varepsilon}}{\sqrt{n\hat{\sigma}^2 \hat{\mathbf{\Theta}}_j^\top \hat{\Sigma}\hat{\mathbf{\Theta}}_j}} - \frac{\hat{\mathbf{\Theta}}_j^\top \mathbb{X}^\top \boldsymbol{\varepsilon}}{\sqrt{n\sigma^2\Theta_{j,j}}}\Big| \\
=& \Big|\frac{\hat{\mathbf{\Theta}}_j^\top \mathbb{X}^\top \boldsymbol{\varepsilon}}{\sqrt{n}}\Big| \cdot \Big|\frac{1}{\sqrt{\hat{\sigma}^2 \hat{\mathbf{\Theta}}_j^\top \hat{\Sigma}\hat{\mathbf{\Theta}}_j}} - \frac{1}{\sqrt{\sigma^2\Theta_{j,j}}}\Big| \\
=& I_{1,1} \cdot I_{1,2}.
\end{aligned} \tag{S.20}$$

As

$$\frac{\hat{\mathbf{\Theta}}_j^\top \mathbb{X}^\top \boldsymbol{\varepsilon}}{n} = \frac{(\mathbb{X}_j - \mathbb{X}_{D_j}\hat{\boldsymbol{\gamma}}_j)^\top \boldsymbol{\varepsilon}}{n\hat{\tau}_j^2} = \frac{\mathbb{X}_j^\top (I - P_{D_j})\boldsymbol{\varepsilon}}{n\hat{\tau}_j^2},$$

where $P_{D_j} = \mathbb{X}_{D_j}(\mathbb{X}_{D_j}^\top \mathbb{X}_{D_j})^{-1}\mathbb{X}_{D_j}^\top$, and by (S.9), we have $\hat{\tau}_j^2 = \tau_j^2 + o_p(1)$. To bound $I_{1,1}$, we need to bound

$$\frac{\mathbb{X}_j^\top (I - P_{D_j})\boldsymbol{\varepsilon}}{n}.$$

By some calculation, we have

$$X_{i,j}|\boldsymbol{X}_{i,D_j} \sim N(-\boldsymbol{X}_{i,D_j}^\top \Theta_{D_j,j}\Theta_{j,j}^{-1}, \Theta_{j,j}^{-1}),$$

which means that

$$X_{i,j} = -X_{i,D_j}^\top \Theta_{D_j,j}\Theta_{j,j}^{-1} + \Theta_{j,j}^{-1/2} U_{i,1},$$

where $U_{i,1} \sim N(0,1)$.

Let $A^{(j)} = I - P_{D_j}$ and $\boldsymbol{U}_1 = (U_{1,1}, \ldots, U_{n,1})^\top$. Then $A^{(j)}$ can be expressed as $A^{(j)} = \sum_{i=1}^n s_i u_i u_i^\top$, where s_i is the singular value of $A^{(j)}$, u_i is the eigenvector corresponding to the eigenvalue s_i. Then, by Bernstein-type inequality for centered subexponential random variables (Proposition 5.16 in Vershynin (2010)), we have

$$\begin{aligned} & P\left(|\frac{1}{n}\mathbb{X}_j^\top (I - P_{D_j})\boldsymbol{\varepsilon}| \geq t \middle| \mathbb{X}_{D_j}\right) \\ & = P\left(|\sum_{i=1}^n s_i \langle u_i, \boldsymbol{\varepsilon}\rangle \langle u_i, \boldsymbol{U}_1\rangle| \geq \Theta_{j,j}^{1/2} nt \middle| \mathbb{X}_{D_j}\right) \\ & \leq 2\exp\left(-c\frac{\Theta_{j,j} n^2 t^2}{n - d_j}\right). \end{aligned} \tag{S.21}$$

Taking $t = \sqrt{(n-d_j)\log p/(c\Theta_{j,j}n^2)}$, (S.21) implies that

$$\begin{aligned} & P\left(|\frac{1}{n}\mathbb{X}_j^\top (I - P_{D_j})\boldsymbol{\varepsilon}| \geq t\right) \\ & \leq E\left(P\left(|\frac{1}{n}\mathbb{X}_j^\top (I - P_{D_j})\boldsymbol{\varepsilon}| \geq t \mid \mathbb{X}_{D_j}\right)\right) \\ & \leq 2p^{-1}. \end{aligned} \tag{S.22}$$

Thus, by (S.22), we have

$$I_{1,1} = \sqrt{\log p}. \tag{S.23}$$

By Theorem 2 and Theorem 3, we have

$$
\begin{aligned}
I_{1,2} =& \left| \frac{1}{\sqrt{\hat{\sigma}^2 \hat{\boldsymbol{\Theta}}_j^\top \hat{\Sigma} \hat{\boldsymbol{\Theta}}_j}} - \frac{1}{\sqrt{\sigma^2 \Theta_{j,j}}} \right| \\
=& \left| \frac{\sqrt{\hat{\sigma}^2 \hat{\boldsymbol{\Theta}}_j^\top \hat{\Sigma} \hat{\boldsymbol{\Theta}}_j} - \sqrt{\sigma^2 \Theta_{j,j}}}{\sqrt{\sigma^2 \Theta_{j,j}} \sqrt{\hat{\sigma}^2 \hat{\Theta}_j^T \hat{\Sigma} \hat{\Theta}_j}} \right| \\
\leq& \frac{|\hat{\sigma}^2 \hat{\boldsymbol{\Theta}}_j^\top \hat{\Sigma} \hat{\boldsymbol{\Theta}}_j - \sigma^2 \Theta_{j,j}|^{1/2}}{\sqrt{\sigma^2 \Theta_{j,j}} \sqrt{\hat{\sigma}^2 \hat{\boldsymbol{\Theta}}_j^\top \hat{\Sigma} \hat{\boldsymbol{\Theta}}_j}} \\
\leq& \frac{|\hat{\sigma}^2 \hat{\boldsymbol{\Theta}}_j^\top \hat{\Sigma} \hat{\boldsymbol{\Theta}}_j - \sigma^2 \hat{\boldsymbol{\Theta}}_j^\top \hat{\Sigma} \hat{\boldsymbol{\Theta}}_j|^{1/2} + |\sigma^2 \hat{\boldsymbol{\Theta}}_j^\top \hat{\Sigma} \hat{\boldsymbol{\Theta}}_j - \sigma^2 \Theta_{j,j}|^{1/2}}{\sqrt{\sigma^2 \Theta_{j,j}} \sqrt{\hat{\sigma}^2 \hat{\boldsymbol{\Theta}}_j^\top \hat{\Sigma} \hat{\boldsymbol{\Theta}}_j}} \\
=& O_p\left(\frac{\sqrt{s_0 \log p}}{\sqrt{n}} \right) + \frac{(d \log p)^{1/4}}{n^{1/4}}.
\end{aligned}
\tag{S.24}
$$

Therefore, by (S.20), (S.23) and (S.24), we conclude that

$$
I_1 = O_p \left(\log p \sqrt{s_0} / \sqrt{n} + (\log p)^{3/4} d^{1/4} / n^{1/4} \right).
$$

By Lemma S.3, conditions (C2) and (C3), it is straightforward to see that

$$
I_2 = O_p \left(d \sqrt{\frac{\log d \log p}{n}} \right).
$$

Then, we complete the proof of equality (S.18). Therefore, by (S.18), (S.28) and condition of Theorem 4, we have

$$
\begin{aligned}
|\tilde{T}_n - \check{T}_n| \leq& \max_{1 \leq j \leq p} |\tilde{T}_j^2 - \check{T}_j^2| \\
\leq& c \max_{1 \leq j \leq p} |\tilde{T}_j| \max_{1 \leq j \leq p} |\tilde{T}_j - \check{T}_j| \\
=& o_p(1).
\end{aligned}
$$

For event B_1, by Lemma S.1 and Theorem 1, we have

$$
\begin{aligned}
\max_{1 \leq j \leq p} |\tilde{T}_j - T_j| =& \max_{1 \leq j \leq p} \frac{\sqrt{n} |(\hat{\Sigma} \hat{\boldsymbol{\Theta}}_j - \boldsymbol{e}_j)^\top (\hat{\boldsymbol{\beta}} - \boldsymbol{\beta})|}{\hat{\boldsymbol{\Theta}}_j^\top \hat{\Sigma} \hat{\boldsymbol{\Theta}}_j} \\
\leq& \max_{1 \leq j \leq p} \frac{\sqrt{n} \|\hat{\Sigma} \hat{\boldsymbol{\Theta}}_j - \boldsymbol{e}_j\|_\infty \|\hat{\boldsymbol{\beta}} - \boldsymbol{\beta})\|_1}{\hat{\boldsymbol{\Theta}}_j^\top \hat{\Sigma} \hat{\boldsymbol{\Theta}}_j} \\
=& O_p(\sqrt{n} s_0 \log p / n).
\end{aligned}
$$

Therefore, by the condition of Theorem 4, we have

$$\begin{aligned}
|\tilde{T}_n - T_n| \leq & \max_{1\leq j\leq p} |\tilde{T}_j^2 - T_j^2| \\
\leq & c \max_{1\leq j\leq p} |\check{T}_j| \max_{1\leq j\leq p} |\tilde{T}_j - T_j| \\
= & O_p(\sqrt{\log p}) O_p(\sqrt{n} s_0 \log p/n) \\
= & o_p(1).
\end{aligned}$$

Lemma S.5 (Bonferroni inequality) *Let $B = \cup_{t=1}^p B_t$. For any integer $k < p/2$, we have*

$$\sum_{t=1}^{2k} (-1)^{t-1} A_t \leq P(B) \leq \sum_{t=1}^{2k-1} (-1)^{t-1} A_t,$$

where $A_t = \sum_{1\leq i_1 < \ldots < i_t \leq p} P(B_{i_1} \cap \ldots \cap B_{i_t})$.

Lemma S.6 *Define $\check{T}_j = \frac{\boldsymbol{\Theta}_j^\top \mathbb{X}^\top \boldsymbol{\varepsilon}}{\sqrt{n\sigma^2 \boldsymbol{\Theta}_j^\top \Sigma \boldsymbol{\Theta}_j}} = \frac{\boldsymbol{\eta}_j^\top \boldsymbol{\varepsilon}}{\sqrt{n\sigma^2/\Theta_{j,j}}}$, $j = 1, \ldots, p$, and $\check{T}_n = \max_j \check{T}_j^2$. Then, for any given $x \in R$,*

$$P(\check{T}_n - 2\log p + \log\log p \leq x) \to \exp\left(-\frac{1}{\sqrt{\pi}} \exp(-x/2)\right), \text{ as } (n,p) \to \infty.$$

The idea of the proof of Lemma S.6 is similar to that used in Ma, Cai and Li (2021)

Proof: Let $x_p = 2\log p - \log\log p + x$. Therefor it suffices to prove

$$P(\check{T}_n \leq x_p) \to \exp\left(-\frac{1}{\sqrt{\pi}} \exp(-x/2)\right).$$

Define $\hat{T}_j = \frac{\sum_{i=1}^n Z_{i,j}}{\sqrt{n\sigma^2/\Theta_{j,j}}}$, $j = 1, \ldots, p$. where $Z_{i,j} = \boldsymbol{\eta}_{i,j}\boldsymbol{\varepsilon}_i I\{|\boldsymbol{\eta}_{i,j}\boldsymbol{\varepsilon}_i| \leq \tau_n\} - E[\boldsymbol{\eta}_{i,j}\boldsymbol{\varepsilon}_i I\{|\boldsymbol{\eta}_{i,j}\boldsymbol{\varepsilon}_i| \leq \tau_n\}]$ for $\tau_n = \log(p+n)$, and $\hat{T}_n = \max_j \hat{T}_j^2$.

Fist, we are going to show that

$$|\hat{T}_n - \check{T}_n| = o(1),$$

with probability at least $1 - O(p^{-c})$ for some constant $c > 0$.

Note that

$$\begin{aligned}
& \max_{1\leq j\leq p} \frac{1}{\sqrt{n}} \sum_{i=1}^n E[|\boldsymbol{\eta}_{i,j}\boldsymbol{\varepsilon}_i| I\{|\boldsymbol{\eta}_{i,j}\boldsymbol{\varepsilon}_i| \geq \tau_n\}] \\
\leq & Cn^{1/2} \max_{i,j} E[|\boldsymbol{\eta}_{i,j}\boldsymbol{\varepsilon}_i| I\{|\boldsymbol{\eta}_{i,j}\boldsymbol{\varepsilon}_i| \geq \tau_n\}] \\
\leq & Cn^{1/2}(n+p)^{-1} \max_{i,j} E[|\boldsymbol{\eta}_{i,j}\boldsymbol{\varepsilon}_i| e^{|\boldsymbol{\eta}_{i,j}\boldsymbol{\varepsilon}_i|}] \qquad \text{(S.25)} \\
\leq & Cn^{1/2}(n+p)^{-1} \max_{i,j} \sqrt{E\left(\boldsymbol{\eta}_{i,j}\boldsymbol{\varepsilon}_i\right)^2 |} \sqrt{E\exp\left(2|\boldsymbol{\eta}_{i,j}\boldsymbol{\varepsilon}_i|\right)} \\
\leq & Cn^{1/2}(n+p)^{-1}.
\end{aligned}$$

Then, on the event $\{\max_{i,j} |\boldsymbol{\eta}_{i,j}\boldsymbol{\varepsilon}_i| \le \tau_n\}$, we have

$$Z_{i,j} = \boldsymbol{\eta}_{i,j}\boldsymbol{\varepsilon}_i - E[\boldsymbol{\eta}_{i,j}\boldsymbol{\varepsilon}_i I\{|\boldsymbol{\eta}_{i,j}\boldsymbol{\varepsilon}_i| \le \tau_n\}],$$

then, by (S.25), we have

$$\begin{aligned}\max_{1\le j\le p} |\hat{T}_j - \check{T}_j| \le& \max_{1\le j\le p} |\frac{1}{\sqrt{n\sigma^2/\Theta_{j,j}}}\sum_{i=1}^n E[\boldsymbol{\eta}_{i,j}\boldsymbol{\varepsilon}_i I\{|\boldsymbol{\eta}_{i,j}\boldsymbol{\varepsilon}_i| \le \tau_n\}]| \\ \le& \max_{1\le j\le p} |\frac{1}{\sqrt{n\sigma^2/\Theta_{j,j}}}\sum_{i=1}^n E[\boldsymbol{\eta}_{i,j}\boldsymbol{\varepsilon}_i I\{|\boldsymbol{\eta}_{i,j}\boldsymbol{\varepsilon}_i| \ge \tau_n\}]| \\ \le& \max_{1\le j\le p} \frac{1}{\sqrt{n\sigma^2/\Theta_{j,j}}}\sum_{i=1}^n E[|\boldsymbol{\eta}_{i,j}\boldsymbol{\varepsilon}_i| I\{|\boldsymbol{\eta}_{i,j}\boldsymbol{\varepsilon}_i| \ge \tau_n\}] \\ \le& C n^{1/2}(n+p)^{-1}.\end{aligned}$$

By Bernstein-type inequality (Proposition 5.16 in Vershynin (2010)),we have

$$P\left(\max_{1\le j\le p} |\hat{T}_j - \check{T}_j| \ge C n^{1/2}(n+p)^{-1}\right) \le P\left(\max_{i,j} |\boldsymbol{\eta}_{i,j}\boldsymbol{\varepsilon}_i| \ge \tau_n\right) = O(p^{-c}). \tag{S.26}$$

Using the same argument, we can prove

$$\begin{aligned}P\left(\max_{1\le j\le p} |\frac{1}{\sqrt{n}}\check{T}_j| > t\right) =& P\left(\max_{1\le j\le p} |\frac{\boldsymbol{\eta}_j^\top \boldsymbol{\varepsilon}}{n\sqrt{\sigma^2/\Theta_{j,j}}}| > t\right) \\ \le& \sum_{j=1}^p P\left(|\frac{\boldsymbol{\eta}_j^\top \boldsymbol{\varepsilon}}{n\sqrt{\sigma^2/\Theta_{j,j}}}| > t\right) \\ \le& 2\sum_{j=1}^p \exp\left[-\min\left(c_1 n t^2, c_2 n t\right)\right].\end{aligned} \tag{S.27}$$

Taking $t = 2\sqrt{\log p/n}/c_1$, we have $P\left(\max_{1\le j\le p} |\check{T}_j| > t\right) = 2p^{-1}$, which implies

$$\max_{1\le j\le p} |\check{T}_j| = O_p(\sqrt{\log p}). \tag{S.28}$$

By (S.26) and (S.28), we have

$$|\hat{T}_n - \check{T}_n| \le \max_{1\le j\le p} |\hat{T}_j^2 - \check{T}_j^2| \le c \max_{1\le j\le p} |\check{T}_j| \max_{1\le j\le p} |\hat{T}_j - \check{T}_j| = o_p(1).$$

Therefor it suffices to prove

$$P(\hat{T}_n \le x_p) \to \exp\left(-\frac{1}{\sqrt{\pi}}\exp(-x/2)\right). \tag{S.29}$$

By Lemma S.5, for any integer $0 < q < p/2$, we have

$$\sum_{l=1}^{2q}(-1)^{l-1}\sum_{1\le j_1<\ldots<j_l\le p} P\left(\bigcap_{k=1}^{l} A_{j_k}\right) \le P(\max_{1\le j\le p}\hat{T}_j^2 \ge x_p) \le \sum_{l=1}^{2q-1}(-1)^{l-1}\sum_{1\le j_1<\ldots<j_l\le p} P\left(\bigcap_{k=1}^{l} A_{j_k}\right), \tag{S.30}$$

where $A_{j_k} = \{\hat{T}_{j_k}^2 \ge x_p\}$. Let $W_{i,j} = \frac{Z_{i,j}}{\sqrt{\sigma^2/\Theta_{j,j}}}$ for $j = 1, \ldots, p$ and $\boldsymbol{W}_i = (W_{i,j_1}, \ldots, W_{i,j_l})^T$ for $i = 1, \ldots, n$. Define $\|\boldsymbol{a}\|_{\min} = \min_{1\le i\le l}|a_i|$ for any vector $\boldsymbol{a} \in R^l$. Then, we have

$$P\left(\bigcap_{k=1}^{l} A_{j_k}\right) = P\left(\|n^{-1/2}\sum_{i=1}^{n}\boldsymbol{W}_i\|_{\min} \ge x_p^{1/2}\right).$$

Then it follows from Theorem 1.1 in Zaitsev (1987) that

$$P\left(\|n^{-1/2}\sum_{i=1}^{n}\boldsymbol{W}_i\|_{\min} \ge x_p^{1/2}\right) \le P\left(\|\boldsymbol{N}_l\|_{\min} \ge x_p^{1/2} - \epsilon_n(\log p)^{-1/2}\right) + c_1 l^{5/2}\exp\left\{-\frac{n^{1/2}\epsilon_n}{c_2 l^3\tau_n(\log p)^{1/2}}\right\}, \tag{S.31}$$

where $c_1 > 0$ and $c_2 > 0$ are constants, $\epsilon_n \to 0$, and $\boldsymbol{N}_l$ is a normal vector with $E(\boldsymbol{N}_l) = 0$ and $Cov(\boldsymbol{N}_l) = Cov(\boldsymbol{W}_1)$. Let $\epsilon_n = \sqrt{(\log p)^5/n}$. Because l is a fixed integer that does not depend on n, p, so we have

$$c_1 l^{5/2}\exp\left\{-\frac{n^{1/2}\epsilon_n}{c_2 l^3\tau_n(\log p)^{1/2}}\right\} = O(p^{-c}). \tag{S.32}$$

Combining (S.30), (S.31), (S.32) and Lemma 4 of Cai, Liu, and Xia (2013), we have

$$P(\max_{1\le j\le p}\hat{T}_j^2 \ge x_p) \le \sum_{l=1}^{2q-1}(-1)^{l-1}\sum_{1\le j_1<\ldots<j_l\le p} P\left(\|\boldsymbol{N}_l\|_{\min} \ge x_p^{1/2} - \epsilon_n(\log p)^{-1/2}\right) + o(1) \le \sum_{l=1}^{2q-1}(-1)^{l-1}\frac{1}{l!}\left(\frac{1}{\sqrt{\pi}}\exp(-x/2)\right)^l(1+o(1)). \tag{S.33}$$

Similarly, we can derive

$$P(\max_{1\le j\le p}\hat{T}_j^2 \ge x_p) \ge \sum_{l=1}^{2q}(-1)^{l-1}\sum_{1\le j_1<\ldots<j_l\le p} P\left(\|\boldsymbol{N}_l\|_{\min} \ge x_p^{1/2} + \epsilon_n(\log p)^{-1/2}\right) + o(1) \ge \sum_{l=1}^{2q}(-1)^{l-1}\frac{1}{l!}\left(\frac{1}{\sqrt{\pi}}\exp(-x/2)\right)^l(1+o(1)). \tag{S.34}$$

Combining (S.33) and (S.34), we can obtain (S.29) and the proof is complete.

Proof of Theorem 4:

This is completed directly from Lemma S.4 and Lemma S.6.

2 Web Appendix B: Sensitivity analysis

We generate datasets similar to Example 1 and Example 2. To assess the effect of inaccurate or incomplete network information, similar to Zhao and Shojaie (2016), we consider variants of the proposed method with incorrectly specified graphs, where a number of randomly selected edges are added or removed. The number of removed or added edges relative to the true graph is $NPE = \{-5, 15, 105, 165\}$ with negative and positive numbers indicating removals and additions of edges, respectively. For example, $NPE = -5$ indicates that in addition to the true graph represented by L, we randomly remove 5 edges in the true graph, and we call the Laplace matrix corresponding to this wrong graph structure L_{-5}. On the other hands, $NPE = 15$ indicates that in addition to the true edges in L, we also randomly add 15 wrong edges to L, and we call the Laplace matrix corresponding to this wrong graph structure L_{15}. Analogously, L_{105} and L_{165} indicate $NPE = 105$ and $NPE = 165$, respectively.

We define $GCDL_{-5}$, which is the same as the proposed method $GCDL$, except that we replace the true Laplacian matrix L with L_{-5}. $GCDL_{15}$, $GCDL_{105}$ and $GCDL_{165}$ are defined analogously. For convenience in the description, denote by $\hat{b}_j^{GCDL_{-5}}$, $\hat{b}_j^{GCDL_{15}}$, $\hat{b}_j^{GCDL_{105}}$ and $\hat{b}_j^{GCDL_{165}}$ the estimators of β_j based on the method $GCDL_{-5}$, $GCDL_{15}$, $GCDL_{105}$ and $GCDL_{165}$, respectively. According to Theorem 2, the 95% confidence interval $CI_j^{GCDL_{-5}}$ of β_j based on the $GCDL_{-5}$ can be constructed as follows:

$$CI_j^{GCDL_{-5}} = \hat{b}_j^{GCDL_{-5}} \pm \frac{1.96}{\sqrt{n}} \hat{\sigma}_1 \sqrt{{\hat{\Theta}^1}_j^T \hat{\Sigma} {\hat{\Theta}^1}_j},$$

where ${\hat{\Theta}^1}_j$ is the jth column of $\hat{\Theta}^1$, and $\hat{\Theta}^1$ is the estimator of Θ that is the same as $\hat{\Theta}$ except for the wrong Laplacian matrix L_{-5} being used. Similarly, $\hat{\sigma}_1^2$ is the estimator of σ^2 by using L_{-5}. $CI_j^{GCDL_{15}}$, $CI_j^{GCDL_{105}}$ and $CI_j^{GCDL_{165}}$ are defined analogously.

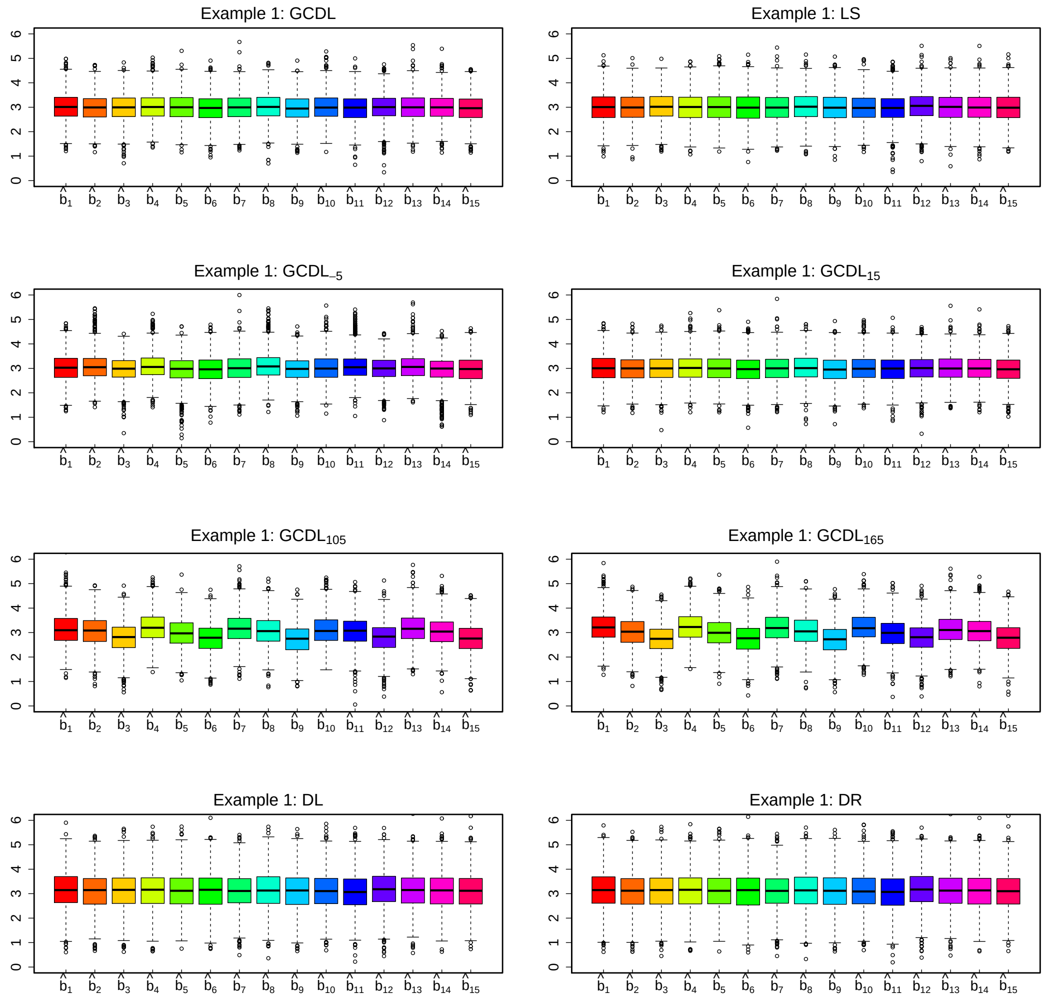

Figure 1: The estimator of $\boldsymbol{\beta}_j, j = 1, \ldots, 15$ (Non-zero coefficients) by eight methods: $GCDL$, $GCDL_{-5}$, $GCDL_{15}$, $GCDL_{105}$, $GCDL_{165}$, LS, DL, and DR.

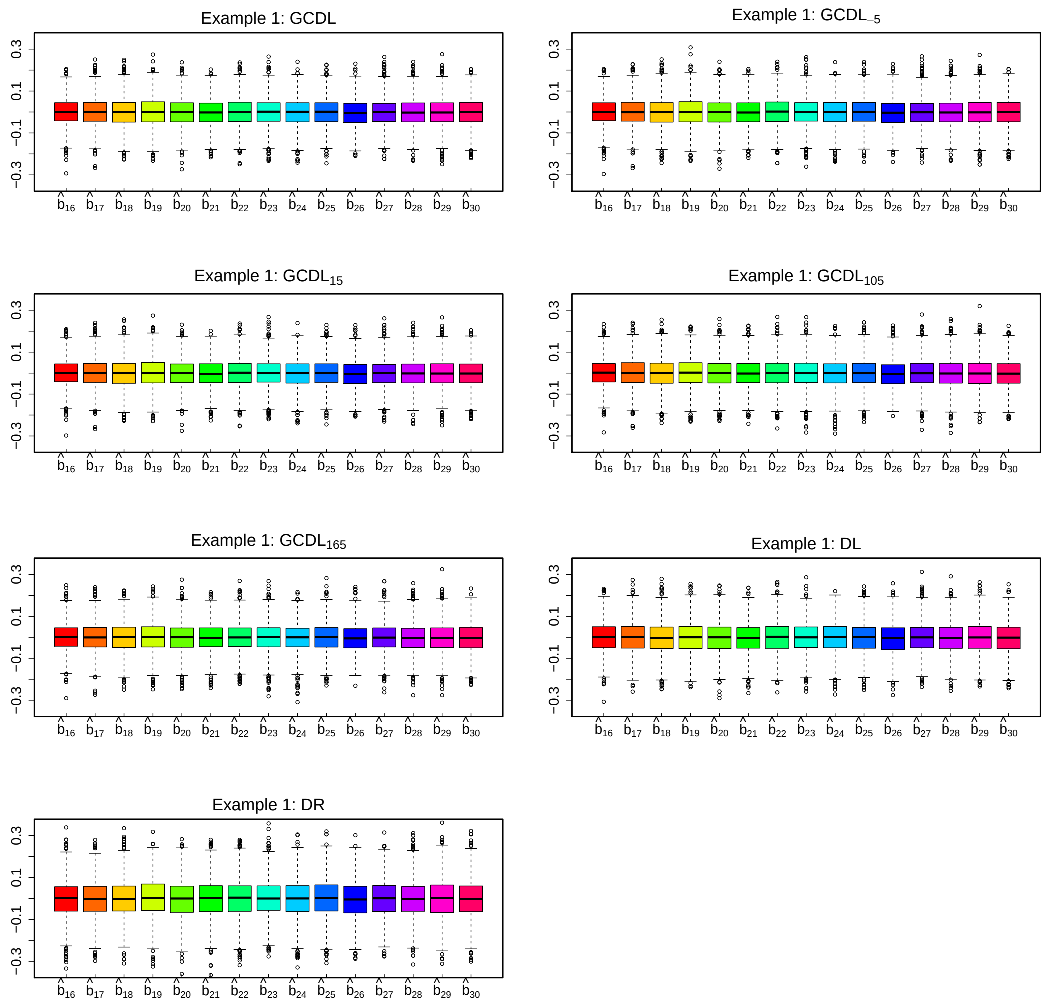

Figure 2: The estimator of $\boldsymbol{\beta}_j, j = 16, \ldots, 30$ (Zero coefficients) by seven methods: $GCDL$, $GCDL_{-5}$, $GCDL_{15}$, $GCDL_{105}$, $GCDL_{165}$, DL, and DR.

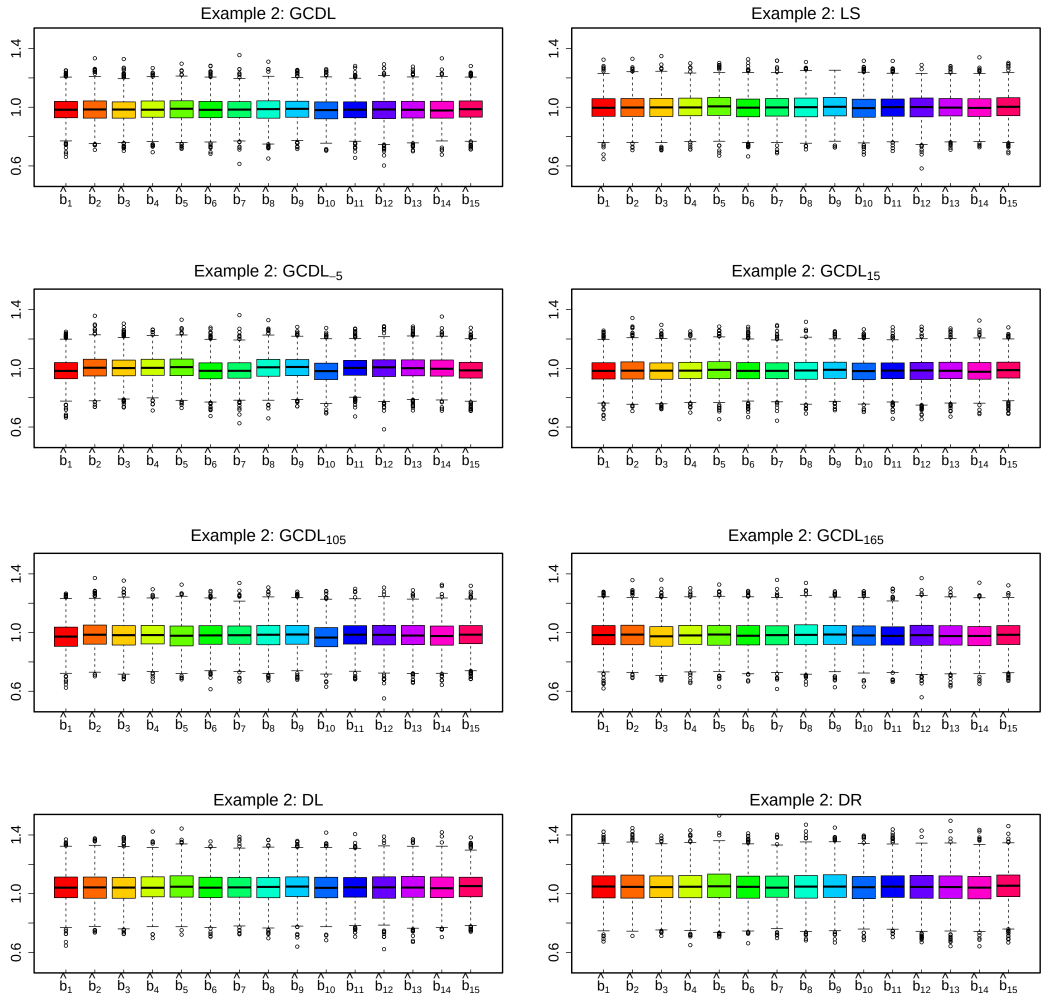

Figure 3: The estimator of $\boldsymbol{\beta}_j, j = 1, \ldots, 15$ (Non-zero coefficients) by eight methods: $GCDL$, $GCDL_{-5}$, $GCDL_{15}$, $GCDL_{105}$, $GCDL_{165}$, LS, DL, and DR.

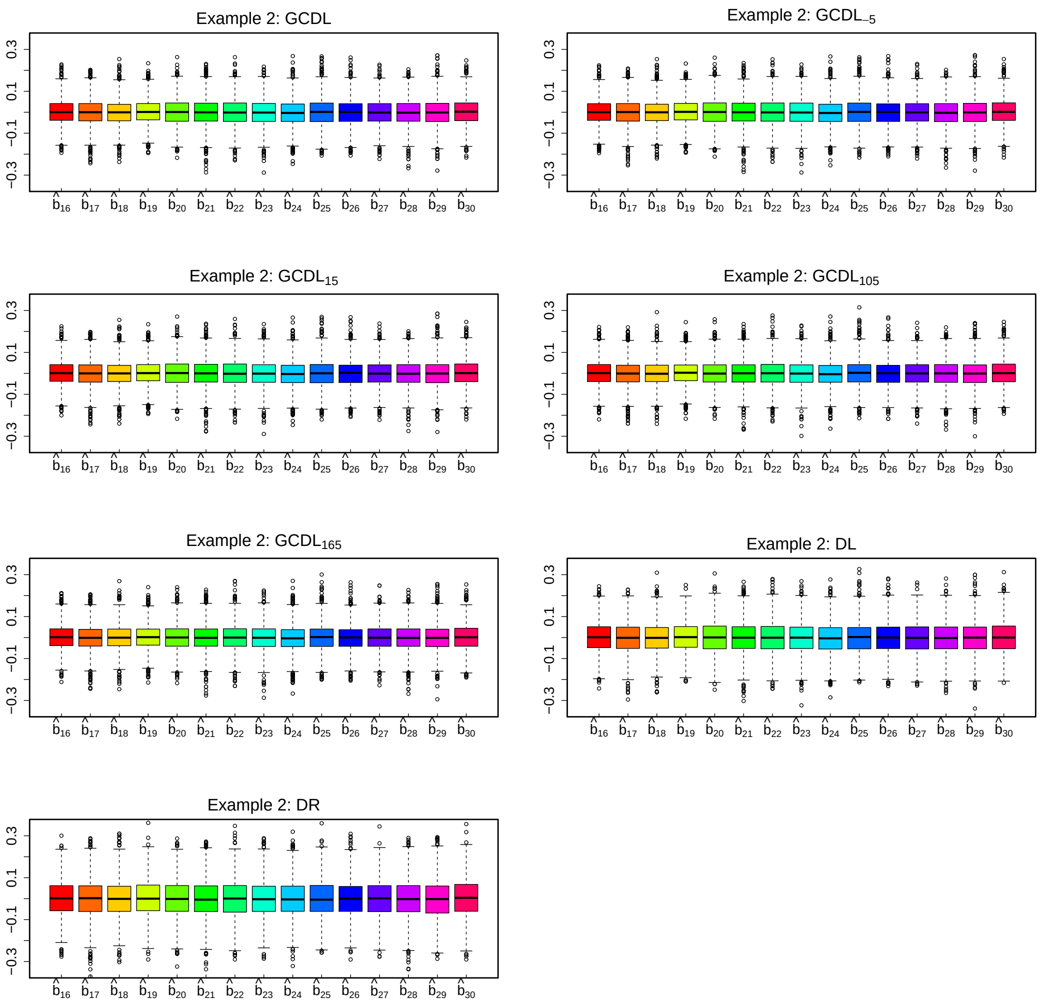

Figure 4: The estimator of $\boldsymbol{\beta}_j, j = 16, \ldots, 30$ (Zero coefficients) by seven methods: $GCDL$, $GCDL_{-5}$, $GCDL_{15}$, $GCDL_{105}$, $GCDL_{165}$, DL, and DR.

Now, we examine the consistency of $\hat{b}_j$. For the non-zero coefficients incorporating the correct graphical structure as conducted in Example 1 and Example 2, Figure 1 and Figure 3 indicate that the proposed method GCDL is comparable with Least Squares (LS). For the zero coefficients, from Figure 2 and Figure 4, it can be found that the the estimators of zero coefficients perform well consistently for all settings even we use the misspecified graph structure.

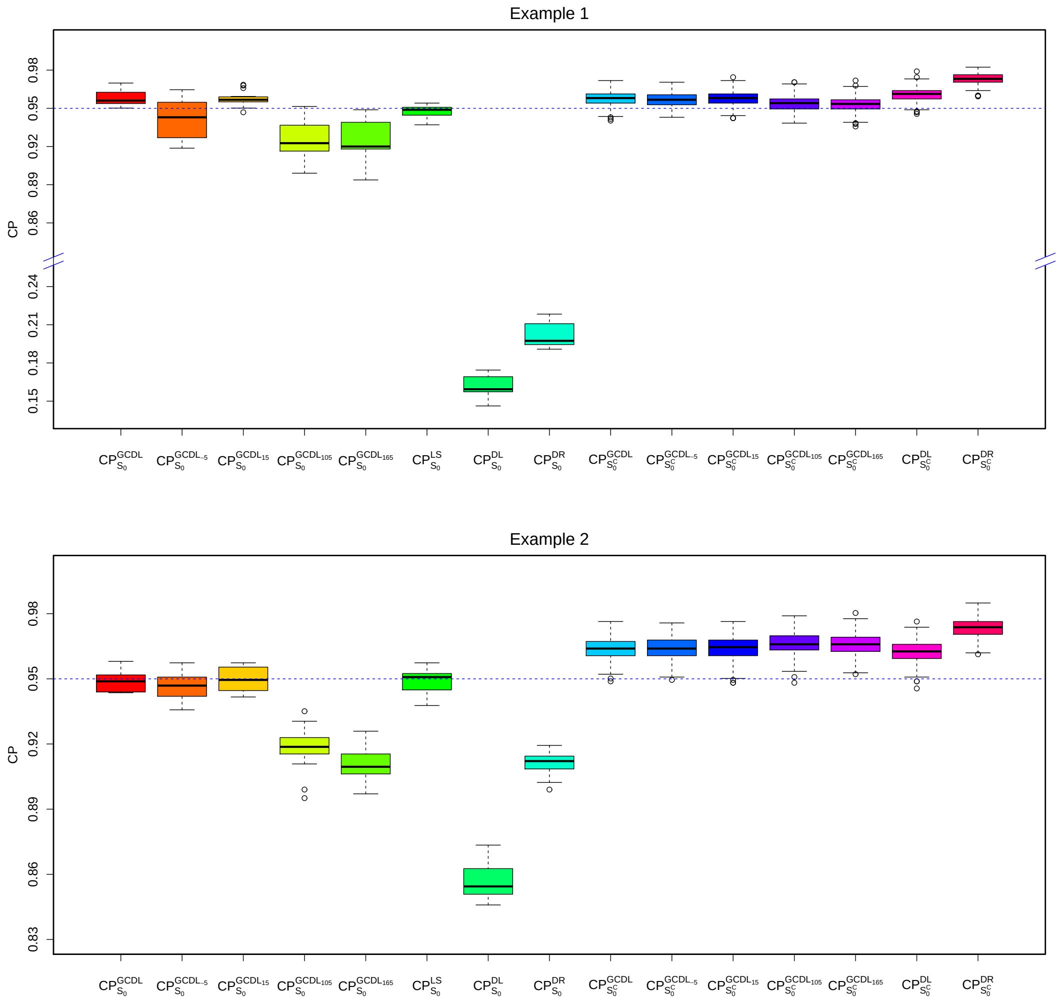

Figure 5: Coverage probabilities computed by eight methods: $GCDL$, $GCDL_{-5}$, $GCDL_{15}$, $GCDL_{105}$, $GCDL_{165}$, LS, DL, and DR. The y-axis in the up panel is truncated.

We compute the empirical coverage probability $CP_j^{GCDL_{-5}} = P_n(\beta_j \in CI_j^{GCDL_{-5}})$, where P_n denotes the empirical probability based on 1000 realizations. The coverage probabilities $CP_j^{GCDL_{15}}$, $CP_j^{GCDL_{105}}$ and $CP_j^{GCDL_{165}}$ are defined analogously. Also, we provide the average lengths $AL_j^{GCDL_{-5}}$, $AL_j^{GCDL_{15}}$, $AL_j^{GCDL_{105}}$ and $AL_j^{GCDL_{165}}$ of the confidence intervals $CI_j^{GCDL_{-5}}$, $CI_j^{GCDL_{15}}$, $CI_j^{GCDL_{105}}$ and $CI_j^{GCDL_{165}}$, respectively, for $j = 1, \ldots, p$. Define $CP_{S_0}^{GCDL_{-5}} = \{CP_j^{GCDL_{-5}} : j \in S_0\}$. $CP_{S_0^c}^{GCDL_{-5}}$, $CP_{S_0}^{GCDL_{15}}$, $CP_{S_0^c}^{GCDL_{15}}$, $CP_{S_0}^{GCDL_{105}}$, $CP_{S_0^c}^{GCDL_{105}}$, $CP_{S_0}^{GCDL_{165}}$, and $CP_{S_0^c}^{GCDL_{165}}$ are defined analogously.

We summarize the results for the coverage probabilities and interval lengths by taking

averages across coordinates on S_0 and S_0^c; that is, we define

$$AveCP_{S_0} = \frac{1}{s_0}\sum_{j\in S_0} CP_j, \quad AveCP_{S_0^c} = \frac{1}{p-s_0}\sum_{j\in S_0^c} CP_j$$

and

$$AveAL_{S_0} = \frac{1}{s_0}\sum_{j\in S_0} AL_j, \quad AveAL_{S_0^c} = \frac{1}{p-s_0}\sum_{j\in S_0^c} AL_j.$$

When the misspecified graphical structure is present, Figure 1 and Figure 3 indicate that the estimated non-zero coefficients by the proposed method may be affected. Figure 5 and Table 1 demonstrate that coverage probabilities from the proposed method become worse when we randomly remove or add edges. But the proposed method performs much better than the desparsified LASSO (DL) and desparsified ridge (DR) methods. Thus, even when the misspecified graph structure is present, the proposed method performs much better than the DL and DR methods, especially in the case of highly correlated predictors.

Table 1: Average coverage probabilities and interval lengths

		$AveCP_{S_0}$	$AveCP_{S_0^c}$	$AveAL_{S_0}$	$AveAL_{S_0^c}$
Example 1	$GCDL$	0.9578	0.9575	2.3006	0.2788
	$GCDL_{-5}$	0.9412	0.9567	2.0917	0.2784
	$GCDL_{15}$	0.9578	0.9576	2.2979	0.2785
	$GCDL_{105}$	0.9260	0.9537	2.3047	0.2795
	$GCDL_{165}$	0.9255	0.9532	2.3024	0.2792
	LS	0.9478	—	2.3693	—
	DL	0.1618	0.9609	0.3311	0.3125
	DR	0.2017	0.9733	0.4090	0.4031
Example 2	$GCDL$	0.9485	0.9639	0.3424	0.2782
	$GCDL_{-5}$	0.9463	0.9641	0.3286	0.2781
	$GCDL_{15}$	0.9495	0.9642	0.3419	0.2779
	$GCDL_{105}$	0.9176	0.9660	0.3421	0.2781
	$GCDL_{165}$	0.9108	0.9660	0.3422	0.2782
	LS	0.9486	—	0.3531	—
	DL	0.8573	0.9625	0.3295	0.3151
	DR	0.9113	0.9736	0.4193	0.4040

For the nonzero regression coefficients, Table 1 indicates that the average coverage probability decreases with the increase of error edge, but it is better than DL and DR methods. Compared with the case of adding the wrong edge, removing the edge of the true graph structure will reduce the average confidence interval. This phenomenon shows that removing the edge of the true graph structure may affects the estimator $\hat{\Theta}$.

For the all regression coefficients, the average confidence intervals obtained by methods GCDL, $GCDL_{15}$, $GCDL_{105}$, and $GCDL_{165}$ are almost the same. That is, our proposed method of estimating $\hat{\Theta}$ is almost unaffected by adding graph edges. This phenomenon shows that adding graph edges mainly affects the desparsified estimation of non-zero coefficients.

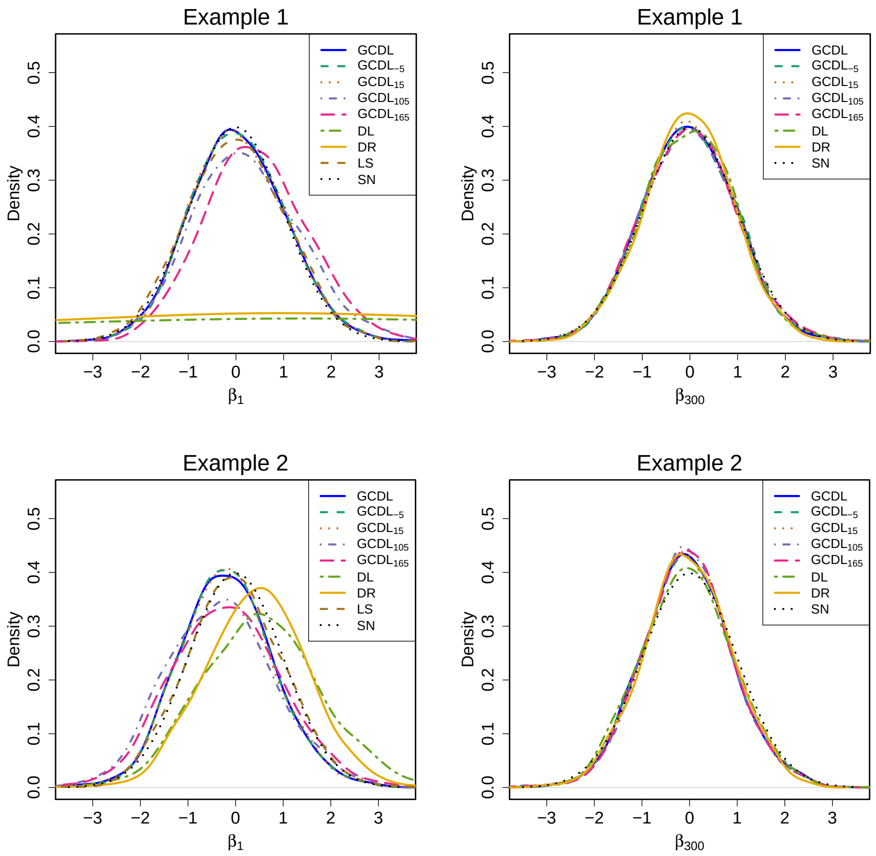

Figure 6: Empirical densities of $\hat{b}_j^{GCDL}$, $\hat{b}_j^{GCDL_{-5}}$, $\hat{b}_j^{GCDL_{15}}$, $\hat{b}_j^{GCDL_{105}}$, $\hat{b}_j^{GCDL_{165}}$, $\hat{b}_j^{DL}$, $\hat{b}_j^{DR}$, and $\hat{\beta}_1^{LS}$ for $j = 1$ and 300 based on 1000 realizations. "SN" means standard normal.

We empirically verify the asymptotic normality of $\hat{b}_j$ in Theorem 2 by different methods. We compute $\hat{b}_j$, $j = 1, \ldots, p$, based on 1000 realizations, and then plot their empirical densities. Figure 6 only shows the results for $j = 1$ and 300.

In both settings, Figure 6 confirms the asymptotic normality of the proposed method. Specifically, after centering and standardization, the distribution of $\hat{b}_j^{GCDL}$ is close to the standard normal distribution for $j = 1$ and 300, but the distribution of $\hat{b}_j^{DL}$ and $\hat{b}_j^{DR}$ are far from the standard normal distribution when $j = 1$. For the $GCDL_{-5}$, $GCDL_{15}$ $GCDL_{105}$

and $GCDL_{165}$ methods, when the misspecified graph structure is present, from Figure 6, it seems that the asymptotic normality of the estimated non-zero coefficient of the proposed method is still valid. And consequently, it can be found that there is little difference between the estimated density curve and the density curve of the standard normal distribution. In addition, Figure 6 indicates again that the estimator of zero coefficient is not sensitive to the misspecified graphical structure.

We verify numerically the asymptotic distribution of T_n. Denote by T_n^{GCDL}, T_n^{DL} and T_n^{DR} the test statistics based on the method GCDL (The proposed method), the desparsified LASSO (DL) and the desparsified ridge (DR), respectively.

Figure 7 confirms the asymptotic distribution of T_n. Specifically, the distribution of T_n^{GCDL} is close to the Gumbel distribution, but the distribution of T_n^{DL} and T_n^{DR} are far from the Gumbel distribution in the case of highly correlated predictors. Also, even if we use the misspecified graph structure, there is little difference between the estimated density curve and the density curve of Gumbel distribution.

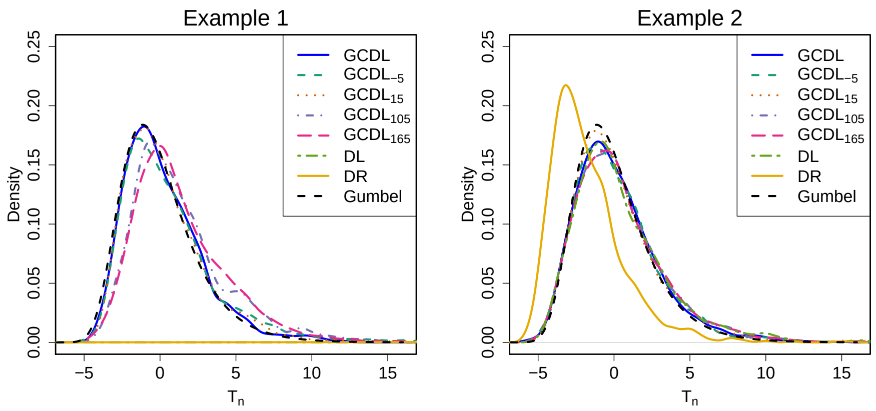

Figure 7: Empirical densities of T_n^{GCDL}, $T_n^{GCDL_{-5}}$, $T_n^{GCDL_{15}}$, $T_n^{GCDL_{105}}$, $T_n^{GCDL_{165}}$, T_n^{DL}, T_n^{DR}, and T_n^{LS} based on 1000 realizations.

Therefore, we conclude from the simulation results that when the graph structure is misspecified,

1. the estimators of zero coefficients by the proposed procedure are insensitive to the misspecified graphical structure.

2. the estimators of non-zero coefficients by the proposed method are affected by the misspecification of the graphical structure. However, the proposed procedure performs much better than the desparsified LASSO and desparsified ridge methods.

3 Web Appendix C: Verification of the conditions used for theoretical guarantees based on the real data

In this section, we provide some empirical evidence (based on the real data analysis) to support the conditions used for the theoretical guarantees. In the real data, the sample size is $n = 170$, and the number of predictors is $p = 70$. A total of 11 genes were identified by the proposed method (see details in Section 4). We refit a linear model by regressing the response Y on the selected genes (denoted by $\boldsymbol{X}_{se} = (gene1, \cdots, gene11)^\top$), that is,

$$Y = \alpha + \boldsymbol{X}_{se}^\top \boldsymbol{\beta}_{se} + \varepsilon, \tag{S.1}$$

where $\boldsymbol{\beta}_{se} \in \mathbb{R}^{11}$ is an unknown parameter. Then, we used the classical F-test statistic to test whether β_{se} is zero, that is,

$$H_0 : \boldsymbol{\beta}_{se} = 0 \leftrightarrow H_1 : \boldsymbol{\beta}_{se} \neq 0.$$

The p-value of the F test is 2.299×10^{-6}, which provides strong evidence to reject the null hypothesis and concludes the significant relationship between the selected genes and the response Y. Compared with the total number of genes ($p = 70$), only 11 genes was selected and fitted, which implies that the condition of sparsity assumption (C5) is satisfied.

Theoretically, the Bernstein-Type inequality requires the assumption that $X_{i,j}$ and ε_i satisfy the sub-gaussian condition. In real data, the sample $X_{i,j}$ is bounded, thus, it conforms to the sub-gaussian assumption. We can verify that ε_i is Guassian (then is sub-gaussian) in the real data. Figure 8 depicts the Q-Q plot for the residuals fitted with linear model (S.1). It can be seen from Figure 8 that the residuals are normally distributed. We also conducted the Shapiro-Wilk test for the residuals with a p-value of 0.0659, which implies that the error term ε is Gaussian. Thus, for any $t \geq 0$, by Bernstein-type inequality for

centered sub-exponential random variables (Proposition 5.16 in Vershynin (2010)), we have

$$\begin{aligned} P(\|\mathbb{X}^\top \boldsymbol{\varepsilon}\|_\infty / n \geq t) =& P(\|\mathbb{X}^\top \boldsymbol{\varepsilon}\|_\infty \geq nt) \\ \leq& \sum_{j=1}^{p} P(|\sum_{i=1}^{n} X_{i,j}\varepsilon_i| > nt) \\ \leq& 2p \exp\left(-\min(c_1 nt^2, c_2 nt)\right) \\ =& 2\exp\left(-\min(c_1 nt^2, c_2 nt) + \log p\right). \end{aligned} \tag{S.2}$$

Let $t = \sqrt{\frac{2\log p}{c_1 n}}$, then, we have

$$P(\|\mathbb{X}^\top \boldsymbol{\varepsilon}\|_\infty / n \geq t) \leq 2p^{-1}, \tag{S.3}$$

which implies that Condition (C3) holds.

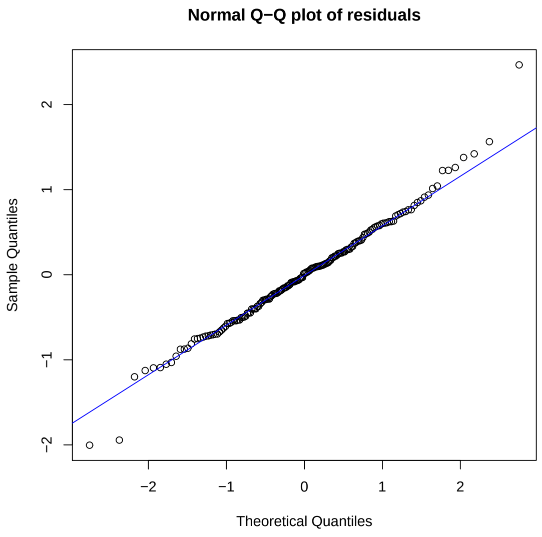

Figure 8: The QQ plot of residuals.

To derive the statistical properties of $\hat{\boldsymbol{\beta}}$, the bound $\|\mathbb{X}^\top \boldsymbol{\varepsilon}\|_\infty / n$ is required. From inequality (S.3), we have

$$\|\mathbb{X}^\top \boldsymbol{\varepsilon}\|_\infty / n = O_p(\sqrt{\log p / n}).$$

Thus, it is mild to impose the assumption $\lambda_1 \asymp \sqrt{\log p / n}$ in Condition (C2), which was used by Bickel, Ritov, and Tsybakov (2009) and Hebiri and Van De Geer (2011), and among many others.

The tuning parameter λ_2 is selected by CV. The selected tuning parameter λ_2 is 0.01131, which approximates $0.10\sqrt{\log p/n} = 0.0158$ (close to the theoretical tuning parameter $\lambda_2 \approx 0.1\sqrt{\log p/n}/\|L\beta\|_\infty$). This condition was imposed by Hebiri and Van De Geer (2011), and among others. Based on the real data, the smallest eigenvalue of $K_n = \hat{\Sigma} + \lambda_2 L$ is 0.159, which combining $p < n$ in the real data, implies that Condition (C1) holds for any $\tilde{\boldsymbol{\beta}} \in R^p$. In addition, the largest and smallest eigenvalue of $\hat{\Sigma}$ is 26.3122 and 0.0005, respectively, implying that Conditions (C4) and (C6) hold.

In summary, all conditions can be checked and verified in the real data set analyzed in this paper, which provides empirical support for the results.

4 Web Appendix D: Additional Tables and Figures

Table 2: The 90% average coverage probabilities and interval lengths.

		$AveCP_{S_0}$	$AveCP_{S_0^c}$	$AveAL_{S_0}$	$AveAL_{S_0^c}$
Example 1	GCDL	0.9142	0.9144	1.9305	0.2338
	LS	0.8967	—	1.9921	—
	DL	0.1368	0.9168	0.2778	0.2620
	DR	0.1727	0.9367	0.3424	0.3376
Example 2	GCDL	0.9011	0.9279	0.2878	0.2337
	LS	0.8956	—	0.2971	—
	DL	0.7792	0.9197	0.2774	0.2649
	DR	0.8466	0.9371	0.3519	0.3387

Table 3: The 99% average coverage probabilities and interval lengths.

		$AveCP_{S_0}$	$AveCP_{S_0^c}$	$AveAL_{S_0}$	$AveAL_{S_0^c}$
Example 1	GCDL	0.9913	0.9907	3.0231	0.3661
	LS	0.9886	—	3.1196	—
	DL	0.2164	0.9933	0.4350	0.4104
	DR	0.2672	0.9963	0.5363	0.5287
Example 2	GCDL	0.9884	0.9919	0.4507	0.3660
	LS	0.9889	—	0.4652	—
	DL	0.9449	0.9931	0.4344	0.4149
	DR	0.9736	0.9962	0.5511	0.5304

Table 4: The 90% average coverage probabilities and interval lengths.

		$AveCP_{S_0}$	$AveCP_{S_0^c}$	$AveAL_{S_0}$	$AveAL_{S_0^c}$
Example 3	AGCDL	0.8982	0.8926	1.9221	0.2376
	LS	0.9013	—	1.9846	—
	GCDL	0.8982	0.8933	1.9221	0.9709
	DL	0.1695	0.9164	0.3245	0.2728
	DR	0.8368	0.9119	0.9162	0.6825

Table 5: The 99% average coverage probabilities and interval lengths.

		$AveCP_{S_0}$	$AveCP_{S_0^c}$	$AveAL_{S_0}$	$AveAL_{S_0^c}$
Example 3	AGCDL	0.9885	0.9888	3.0100	0.3721
	LS	0.9889	—	3.1078	—
	GCDL	0.9885	0.9885	3.0100	1.5205
	DL	0.2612	0.9933	0.5081	0.4273
	DR	0.9628	0.9926	1.4348	1.0689

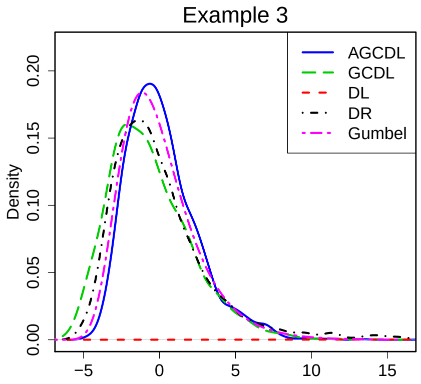

Figure 9: Empirical densities of T_n^{AGCDL}, T_n^{GCDL}, T_n^{DL} and T_n^{DR} based on 1000 realizations.

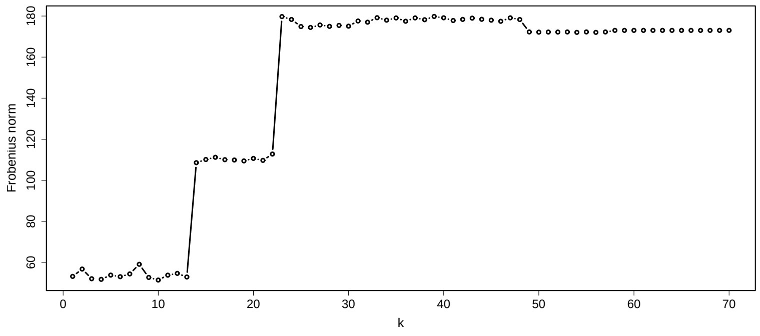

Figure 10: Frobenius norm of $\hat{\Theta}\hat{\Sigma} - I$.

5 Web Appendix E: Sensitivity analysis of the weights in the graph Laplacian matrix L

To check the sensitivity of the proposed method when we use weights in the graph Laplacian matrix L, we construct the Laplacian matrix L according to the graph with all edge weights generated from uniform distribution $U(0.5, 1)$. More specifically, the graph Laplacian matrix L is defined as

$$L_{(u,v)} \triangleq \begin{cases} d_u & \text{if } u = v, \\ -w(u,v) & \text{if } u \text{ and } v \text{ are connected}, \\ 0 & \text{otherwise}, \end{cases}$$

with $w(u,v) \geq 0$, where $w(u,v)$ is the weight of edge $e = (u \backsim v)$ generated from uniform distribution $U(0.5, 1)$, and $d_u = \sum_{v \sim u} w(u,v)$ is the degree of node u. We denote the

proposed GCDL method with the new definition of the Laplacian matrix as WGCDL.

Table 6: Average coverage probabilities and interval lengths

		$AveCP_{S_0}$	$AveCP_{S_0^c}$	$AveAL_{S_0}$	$AveAL_{S_0^c}$
Example 1	GCDL	0.9561	0.9568	2.3034	0.2790
	WGCDL	0.9540	0.9564	2.3020	0.2789
	LS	0.9468	—	2.3745	—
	DL	0.1643	0.9610	0.3303	0.3120
	DR	0.2036	0.9733	0.4078	0.4024
Example 2	GCDL	0.9478	0.9636	0.3431	0.2788
	WGCDL	0.9470	0.9636	0.3430	0.2786
	LS	0.9463	—	0.3541	—
	DL	0.8565	0.9627	0.3300	0.3155
	DR	0.9104	0.9736	0.4198	0.4044

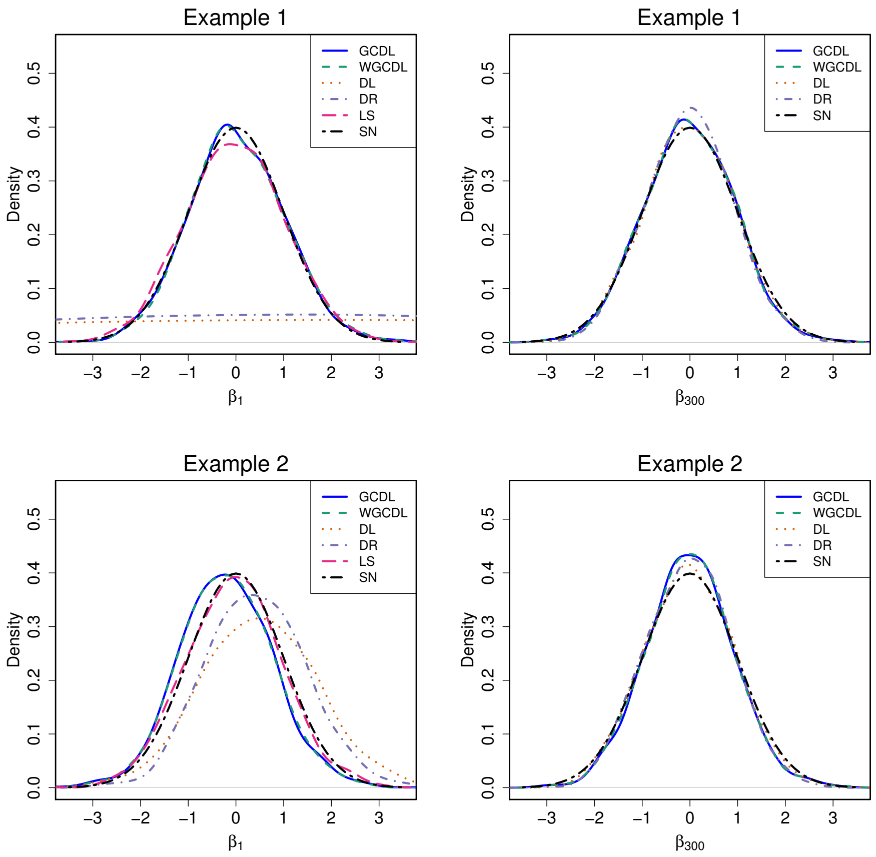

Figure 11: Empirical densities of $\hat{b}_j^{GCDL}$, $\hat{b}_j^{WGCDL}$, $\hat{b}_j^{DL}$, $\hat{b}_j^{DR}$, and $\hat{\beta}_1^{LS}$ for $j = 1$ and 300 based on 1000 realizations. "SN" means standard normal.

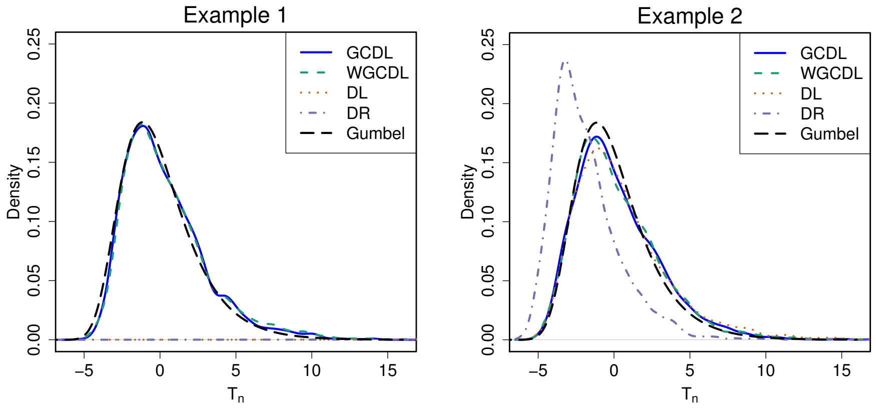

Figure 12: Empirical densities of T_n^{GCDL}, T_n^{WGCDL}, T_n^{DL} and T_n^{DR} based on 1000 realizations.

We generate data as in Examples 1 and 2. Table 6 summarizes the average coverage probabilities and interval lengths for both zero and non-zero regression coefficients in these examples. Notably, both methods, WGCDL and GCDL, exhibit nearly identical average coverage probabilities and confidence intervals. Furthermore, Figures 11 and 12 show that the density functions of both methods coincide. These findings demonstrate the non-sensitivity of the method when using random weights in the graph Laplacian matrix L.

References

Bickel, P. J. , Ritov, Y. , and Tsybakov, A. B. . (2009). Simultaneous analysis of lasso and dantzig selector. *The Annals of Statistics*, **37(4)**, 1705-1732.

Cai, T., Liu, W. and Xia, Y. (2013). Two-sample covariance matrix testing and support recovery in high-dimensional and sparse settings. *Journal of the American Statistical Association*, **108(501)**, 265-277.

Caner, M., and Kock, A. B. (2018). Asymptotically honest confidence regions for high dimensional parameters by the desparsified conservative lasso. *Journal of Econometrics*, **203(1)**, 143-168.

Hebiri, M. and Van De Geer, S. A. (2011). The Smooth-LASSO and other l_1+ l_2-penalized methods. *Electronic Journal of Statistics*. **5**, 1184-1226.

Mikusheva, A. (2007). Uniform inference in autoregressive models. *Econometrica*, **75(5)**, 1411-1452.

Ma, R., Cai, T. and Li, H. (2021) Global and Simultaneous Hypothesis Testing for High-Dimensional Logistic Regression Models, *Journal of the American Statistical Association*, **116:534**, 984-998.

Vershynin, R. (2010). Introduction to the non-asymptotic analysis of random matrices. arXiv preprint arXiv:1011.3027.

Zaitsev, A. Y. . (1987). On the gaussian approximation of convolutions under multidimensional analogues of S.N. bernstein's inequality conditions. *Probability Theory & Related Fields*, **74(4)**, 535-566.

Zhao, S. and Shojaie, A. (2016). A significance test for graph-constrained estimation. *Biometrics*, **72(2)**, 484-493.
